# Biomimetic Magnetite Nanoparticles as Targeted Drug Nanocarriers and Mediators of Hyperthermia in an Experimental Cancer Model

**DOI:** 10.3390/cancers12092564

**Published:** 2020-09-09

**Authors:** Francesca Oltolina, Ana Peigneux, Donato Colangelo, Nausicaa Clemente, Annarita D’Urso, Guido Valente, Guillermo R. Iglesias, Concepcion Jiménez-Lopez, Maria Prat

**Affiliations:** 1 Department of Health Sciences, Università del Piemonte Orientale A. Avogadro, Via Solaroli 17, 28100 Novara, Italy; francesca.oltolina@med.uniupo.it (F.O.); donato.colangelo@med.uniupo.it (D.C.); nausicaa.clemente@med.uniupo.it (N.C.); annarita.durso@uniupo.it (A.D.); 2 Department of Microbiology, University of Granada, Campus Fuentenueva, s/n, 18071 Granada, Spain; apn@ugr.es; 3 Department of Translational Medicine, Università del Piemonte Orientale A. Avogadro, Via Solaroli 17, 28100 Novara, Italy; guido.valente@med.uniupo.it; 4 Department of Applied Physic, University of Granada, Campus Fuentenueva, s/n, 18071 Granada, Spain; iglesias@ugr.es; 5 Centro di Biotecnologie per la Ricerca Medica Applicata (BRMA), Via Solaroli 17, 28100 Novara, Italy; 6 Consorzio Interuniversitario per Biotecnologie (CIB), Località Padriciano 99, 34149 Area di Ricerca, Trieste, Italy; 7 Consorzio Interuniversitario Nazionale per la Scienza e Tecnologia dei Materiali (INSTM), Via Giuseppe Giusti 9, 50121 Firenze, Italy; 8 Consorzio Interuniversitario di Ricerca in Chimica dei Metalli nei Sistemi Biologici (CIRCMSB) Piazza Umberto I 1, 70121 Bari, Italy; 9 Centro Interdipartimentale di Medicina Rigenerativa (CIMeR), Via Montpellier, 1, 00133 Roma, Italy

**Keywords:** magnetic nanoparticles, tumor targeting, cytotoxicity, doxorubicin, hyperthermia

## Abstract

**Simple Summary:**

The application of simultaneous and different strategies to treat cancer appears a promising therapeutic approach. Herein we proposed the application of chemotherapy combined with a magnetic nanocarrier delivery system to an in vitro and an in vivo experimental mammary carcinoma model. Drug-loaded biomimetic magnetic nanoparticle can be directed and concentrated on the tumor cells or site by the apposition of a magnet. Moreover, these nanoparticles can respond to an alternating magnetic field by developing hyperthermia around 43 °C, a temperature at which tumor cells, but not healthy cells, are particularly sensitive and thus induced to death. Indeed, when this nanoformulation is injected in vivo in the tumor site, and hyperthermia is generated, the combined chemo-thermal therapy mediated by these drug-loaded magnetic nanoparticles have a stronger therapeutic benefit compared to that carried out by the chemotherapeutic alone. These nanoformulation and strategy are thus promising tools for translational applications in cancer therapy.

**Abstract:**

Biomimetic magnetic nanoparticles mediated by magnetosome proteins (BMNPs) are potential innovative tools for cancer therapy since, besides being multifunctional platforms, they can be manipulated by an external gradient magnetic field (GMF) and/or an alternating magnetic field (AMF), mediating targeting and hyperthermia, respectively. We evaluated the cytocompatibility/cytotoxicity of BMNPs and Doxorubicin (DOXO)-BMNPs in the presence/absence of GMF in 4T1 and MCF-7 cells as well as their cellular uptake. We analyzed the biocompatibility and in vivo distribution of BMNPs as well as the effect of DOXO-BMNPs in BALB/c mice bearing 4T1 induced mammary carcinomas after applying GMF and AMF. Results: GMF enhanced the cell uptake of both BMNPs and DOXO-BMNPs and the cytotoxicity of DOXO-BMNPs. BMNPs were biocompatible when injected intravenously in BALB/c mice. The application of GMF on 4T1 tumors after each of the repeated (6×) iv administrations of DOXO-BMNPs enhanced tumor growth inhibition when compared to any other treatment, including that with soluble DOXO. Moreover, injection of DOXO-BMNPs in the tumor combined with application of an AMF resulted in a significant tumor weight reduction. These promising results show the suitability of BMNPs as magnetic nanocarriers for local targeted chemotherapy and as local agents for hyperthermia.

## 1. Introduction

With its high burden on lives and being the second most common cause of morbidity and mortality in western countries, cancer represents a major public health problem. Although substantial advancements in therapy have been reached with surgery, chemotherapy, radiotherapy, and immunotherapy, there are still many drawbacks that require novel approaches [1,2]. Chemotherapy is the most common treatment for the majority of tumors, although its limited specificity toward cancer targets is responsible for important severe side effects [3,4,5]. The anthracycline Doxorubicin (DOXO) is one of the most effective chemotherapeutics used for treatment of solid tumors and, in particular, breast cancer. DOXO acts on target cells with different mechanisms. Its interaction with cells begins with passive diffusion through the cell membrane; within the cells, it generates reactive oxygen species (ROS), causing free radical formation and oxidative stress. It can enter the mitochondria, causing DNA damage and energetic stress, by activating the caspase cascade, leading to cell death by apoptosis and triggering autophagy as a consequence of cell energy depletion. Finally, it can translocate into the nucleus, where it intercalates between double-stranded DNA helices and inhibits the enzymes topoisomerases I and II, provoking lethal changes in chromatin structure and the generation of free radicals which, when combined with iron ions, induce oxidative damage to cellular membranes, DNA, and proteins [6,7,8,9]. However, DOXO treatments can induce severe cardiotoxicity due to DOXO accumulation in cardiac tissue [10], which then imposes a narrow therapeutic dose, thus limiting DOXO effectiveness [11]. DOXO efficiency is also compromised by the generation of resistance in cancer cells and by the reduction of drug activity due to physicochemical or physiological conditions in the tumor microenvironment, e.g., hypoxia, acidity, defective vasculature, and the presence of lymphatic vessels [12]. 

It is then clear that new approaches need to be taken to overcome these limitations, so that the effectiveness of DOXO treatments can be increased. One way to increase DOXO efficiency is to optimize selective drug delivery to the tumor site, which could be done by means of nanocarriers that allow external guidance and control. In this context, magnetic nanoparticles (MNPs) offer a series of advantages that make them attractive candidates for this goal. On one hand, as with all nanoparticles (NPs), they can carry high amounts of drugs and provide controlled release of the drug at the tumor site [13,14,15]. NPs allow both passive and active targeting of the tumor. Passive targeting is possible because of the nanometric size of the carrier, thus taking advantage of the enhanced permeability and retention of the microvasculature in the tumor mass [16,17,18]. Active targeting can be achieved both by functionalization of the nanoparticle with probes against tumor-associated markers [15,19,20] or/and, as in the case of MNPs, by the application of a gradient magnetic field (GMF), usually a linear variation in the static magnetic field, which can enhance NP accumulation within the tumor [5,21,22]. Moreover, MNPs can also serve as magnetic hyperthermia (MH) agents, able to induce a local intratumor temperature increase—around 43–46 °C, which is effective against tumor cells—when exposed to an alternating magnetic field (AMF) [15,23,24]. Furthermore, MH also promotes the release or activation of therapeutic molecules coupled to the nanocarriers, thus locally increasing the concentration of the chemotherapeutic drug at the tumor site and prompting the effectiveness of the treatment [15,25,26,27].

For biomedical applications, other than obviously being cytocompatible, MNPs should comply with very specific requirements in order to display the advantages listed above, which are difficult to meet in the already commercialized inorganic ones mainly because of their small size [22].

Firstly, MNPs should be superparamagnetic, i.e., they should show zero magnetization in the absence of a magnetic field. In these conditions, MNPs display only weak reciprocal attractive magnetic interactions that keep them well dispersed, avoiding aggregation due to magnetic dipole particle interaction. Since their size is small (<100 nm), all MNPs behave as single magnetic domains and, thus, are randomly oriented in the absence of an external magnetic field. However, they rapidly rotate to align their magnetic moments to the external field once the external field is applied [22], thus promoting a net magnetization responsible for their guidance [28,29]. These NPs can also develop magnetic energy, which is then translated into heat if they are subjected to an efficient AMF. This behavior is governed by Neel and Brown’s relaxation and depends on the frequency and intensity of the applied magnetic field [30,31]. Therefore, the size of the nanoparticle becomes an important parameter to control. An increase in the size of these MNPs would increase their magnetic moment per particle [32], thus increasing targeting efficiency and the heating power generated per particle unit mass in hyperthermia. 

Secondly, the nanocarrier should expose functional groups that allow functionalization. Moreover, ideally, its isoelectric point (iep) is an important parameter which should facilitate stable interactions with loaded moieties at physiological pH while allowing their release at acidic pH conditions found in the tumor microenvironment [14,15]. These properties are shared by the so-called smart nanomaterials [24].

Functionalization may require the addition of a coating; this can be a disadvantage from many points of view: (i) it requires further manipulations, (ii) it can increase the size, (iii) it increases the overall cost of the synthetic procedure, and (iv) it may interfere with the magnetic response of the MNPs [33].

Therefore, the “bottle neck” for clinical use of MNPs is pending upon the production of a good nano-device that serves as a dual platform for drug delivery and hyperthermia. While their production by chemical means is challenging, many of these drawbacks affecting synthetic MNPs are overcome in biomimetic MNPs (BMNPs), for which production is mediated by magnetosome membrane-associated proteins, mimicking magnetosome production by magnetotactic bacteria [32].

In this context, MamC-mediated BMNPs have been recently proposed as cytocompatible, superparamagnetic NPs. In fact, they have demonstrated their potential as promising drug nanocarriers, even when embedded in liposomes [14,15,34], and as hyperthermia agents [15,23,35], which opens the possibility for combined therapy using the same nanoplatform. MamC modulates the nucleation and growth of the crystal by both template and ionotropic effects [15,36] and remains attached to the nanoparticles, forming a nanocomposite of 95 wt% magnetite + 5 wt% MamC. Such control of MamC on magnetite synthesis in vitro results in magnetic nanoparticles of different sizes and morphologies and thus magnetic properties, compared to those of chemically produced ones, and in nanoparticles with novel surface properties. In fact, these BMNPs display larger sizes (approximately 40 nm) compared to most commercial MNPs (≤30 nm) and show (i) a higher blocking temperature while being superparamagnetic at room temperature and (ii) high saturation magnetization, with these features indicating well-structured MNPs with large magnetic moments per particle, although MamC coating of BMNPs could faintly interfere with their magnetic properties [15]. On the other side, MamC protects BMNPs from oxidation and confers new surface properties to the BMNPs due to exposition of the functional groups of the protein. In fact, these BMNPs have an iep at pH 4.4, which allows electrostatic coupling to positively charged molecules such as DOXO at physiological pH and then drug release at acidic pH [14,15]. In addition, this release can be significantly favored under hyperthermia conditions triggered by AMF [15]. The presence of MamC, conferring a highly negative charge, contributes to electrostatic repulsion within particles and thus to their colloidal stability, which, however, is somehow decreased upon loss of free functional groups after functionalization. Indeed, colloidal stability is one of the major problems of NPs in general. BMNPs are thus potentially useful tools for magnetic drug targeting combined with MH for local regional treatment in cancer.

In this study, we investigated the in vitro responses of BMNPs and DOXO-BMNPs to GMF and AMF by using magnetic field strengths and frequencies physiologically tolerable to find the best working conditions. Then, for the first time, we have described the in vivo distribution and biocompatibility of BMNPs after intravenous injection to ensure a possible safe use in vivo. Finally, the in vivo suitability of the use of DOXO-BMNPs for nano-targeted chemotherapy and MH elicitation in a mammary carcinoma experimental model was evaluated.

## 2. Results and Discussion

### 2.1. In Vitro Cytocompatibility of BMNPs in the Absence/Presence of a GMF

The cytocompatibility of any kind of NP is the first parameter to be ascertained before their eventual biomedical in vivo application [37]. BMNPs were fully characterized in previous studies. In particular, X-ray diffraction (XRD) was run to determine the mineralogy of the solid precipitated (>95% magnetite), and TEM and HRTEM were used to determine the mineralogy, the presence of multiple domains, and/or the presence of organic matter inside the crystal. From TEM micrographs, imageJ program was used to determine the size of the crystals, counting over 1000 crystals. The size of the crystallites was also confirmed by XRD. The ζ-potential, thermogravimetry analyses (TGA) and Fourier Transform Infrared spectrometry (FTIR) were used to determine the surface charge of the nanoparticles and to further evidence functionalization. The hydrodynamic radius and stability measurements were performed in order to determine aggregation and colloidal stability. BET specific surface areas was done for surface area determination. The magnetic properties were determined by means of hysteresis cycle, field coolong-zero field cooling (FC-ZFC) curves, and magnetic hyperthermia, i.e., Specific Power Absorption and Intrinsic Loss Power (SAR and ILP) values [14,15,38]. Although still not ideal, these BMNPs have improved colloidal stability compared to MNPs, even if they are larger (36 ± 12 nm) than the latter (<30 nm). After functionalization with DOXO, their size is only slightly increased (of about 4%) [39].

Moreover, BMNPs were already reported to be cytocompatible on many human cell lines originating from tumors of different origins [14,15,23]. Herein, we tested their cytocompatibility on two breast carcinoma cell lines, the mouse 4T1 and the human MCF-7, both in the absence and in the presence of a GMF. Since the production of reactive oxygen species (ROS) is indicative of cellular oxidative stress leading to cytotoxicity, we first analyzed the cytocompatibility of BMNPs by assessing the level of ROS potentially induced in the two mammary carcinoma cell lines.

**Figure 1:**
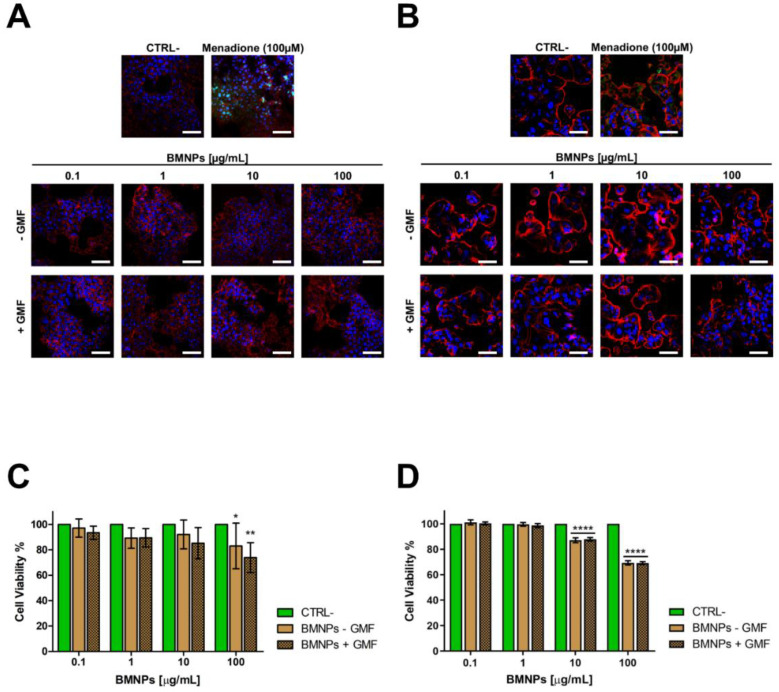
Cytocompatibility of biomimetic magnetic nanoparticles mediated by magnetosome proteins (BMNPs) on 4T1 (**A**,**C**) and MCF-7 (**B**,**D**) cells in the absence/presence of a gradient magnetic field: (**A**,**B**) Analysis of reactive oxygen species (ROS) production in the presence of different concentrations (0.1, 1, 10, and 100 µg/mL) of the BMNPs on 4T1 and on MCF-7 cells in the absence/presence of a gradient magnetic field by confocal microscopy. ROS production (green) was observed only in cells treated with menadione (100 µM), which was used as a positive control. Fixed and permeabilized cells were stained for actin with Tetramethylrhodamine B isothiocyanate (TRITC)-phalloidin (red) and for nuclei with TO-PRO3 (blue). Scale bar: 50 µm. (**C**,**D**) Cell viability assessed in an Methylthiazolyldiphenyl-tetrazolium (MTT) assay after incubation with the same different concentrations of BMNPs for 72 h in presence/absence of a gradient magnetic field (GMF). Differences between groups were assessed by 2-way ANOVA with Dunnett’s multiple comparison test (**** *p* < 0.0001; ** *p* < 0.001; and * *p* < 0.05).

Cells were incubated with increasing concentrations of BMNPs (up to 100 µg/mL) and subjected or not to GMF generated by the application of a neodymium magnet (1.8 kg pull) for 4 h. Under these conditions, no ROS production was detected in any cell line. ROS production was observed as a virtual green color (CellROX^®^ Green Reagent) under confocal microscopy (Figure 1A,B) only in the positive controls where cells were treated with menadione (100 µM), a redox-active quinone that generates superoxides [40], which shows that oxidative stress could be induced in these cells.

The ability of BMNPs to affect signaling pathways linked to cell survival, such as MAPK1/2, and Akt, was also investigated. In fact, the decrease in phosphorylation of these molecules has been linked to the cytotoxicity of some NPs [41,42]. For these experiments, 4T1 cells were incubated for 16 h with different BMNP concentrations up to 100 µg/mL and in the presence/absence of GMF. Protein expression and phosphorylation were analyzed by Western blot (Figure S1). As shown in Figure S1, no significant differences were observed in the levels of phosphorylated and not phosphorylated isoforms of these proteins in any condition tested with respect to untreated controls. The same results were observed for the expression of mTOR, for which expression and level of phosphorylation are under the control of Akt [43]. Taken together, these data show that the presence of BMNPs, either influenced or not by a magnetic field, has no biologic effect on the main signaling pathways controlling cell survival.

Finally, cytocompatibility of BMNPs was assessed by an MTT assay on cells treated with the BMNP concentrations listed above, both in the presence and in the absence of GMF. After the treatments and regardless of the presence/absence GMF, cell viability was always higher than 80% for both cell lines (Figure 1C,D), in agreement with what was observed previously with other cells lines [14,15,23]. Altogether, these data confirm the high cytocompatibility of these BMNPs [44,45].

### 2.2. The Apposition of a GMF Enhances the Interaction of BMNPs with Cells

**Figure 2:**
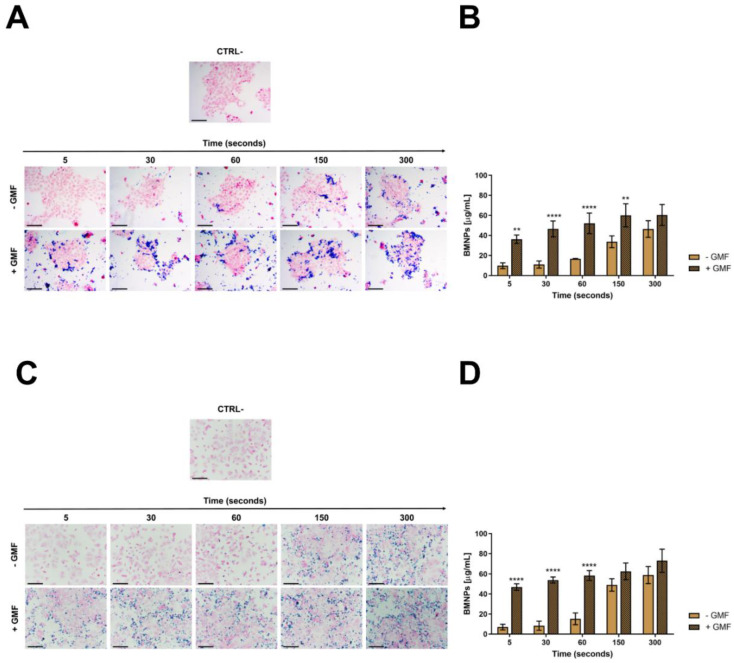
Interaction of BMNPs with 4T1 and MCF-7 cells in the presence/absence of a continuous gradient magnetic field: (**A**,**C**) Images showing BMNPs after Prussian blue staining and nuclear fast red counterstaining; scale bar: 50 µm. (**B**,**D**) Graphs showing the amount of iron associated with the types of cells as quantified with potassium thiocyanate: Cells were incubated with BMNPs (100 µg/mL) for different times (from 5 to 300 s) in the absence (−GMF) and presence (+GMF) of a gradient magnetic field. Untreated cells were used as a negative control. The results (expressed as mean ± SD) were obtained in three independent experiments made in triplicates. Differences between groups were assessed by 2-way ANOVA with Sidak’s multiple comparison test. (**** *p* < 0.0001; ** *p* < 0.001).

Showing the full cytocompatibility of BMNPs in the presence of a GMF, we evaluated whether the apposition of the GMF enhanced the interaction of BMNPs with cells. 4T1 and MCF-7 cells plated on coverslips were incubated for different times with 100 µg/mL BMNPs in the presence or absence of a magnet, were fixed, were washed, and were stained with Prussian blue. When a magnetic field was applied to cells, BMNPs were already clearly visible after 5 s of incubation the first time they were analyzed in both cases of 4T1 and MCF-7 cells, while in the absence of a magnetic field, BMNPs were detectable only and at a very low level after 1 min incubation (Figure 2A,C). In both cases, more BMNPs were detectable as the time of incubation increased, but there was always a significant difference between samples treated with the magnetic plate and not for the times assessed.

In fact, the quantification of iron internalization in the cells also supports these differences (Figure 2B,D). In the absence of GMF, a very low concentration of iron was detected associated with the cells up to 1 min of incubation with BMNPs, and then, this concentration increased after 5 min up to 46 µg/mL for 4T1 cells and up to 59 µg/mL for MCF-7 cells. When the same experiments were performed in the presence of GMF, a significant amount of iron (36.2 µg/mL) was already found associated with 4T1 cells after 5 s of incubation, and this iron concentration increased in a time-dependent way until stabilization after 150 s, reaching a value of 60 µg/mL. An identical behavior was observed for MCF-7 cells in the presence of GMF. In this case, the iron concentration associated with the cells after 5 s of incubation with BMNP was 47 µg/mL, and after 150 s, it was 62.5 µg/mL. For longer periods of incubation, the amount of iron associated with cells increased also when GMF was absent, possibly because of the simple sedimentation of BMNPs on the cell surface. The lack of specific targeting for long periods of incubation under static conditions was already reported for apatite nanoparticles functionalized with a probe recognizing a tumor biomarker expressed at the surface of cancer cells [46,47,48]. It should be considered that these experiments were carried out in vitro in static conditions, while in vivo, GMF was applied in a dynamic situation in which BMNPs, which were circulating in the blood stream, were attracted and retained at the tumor site.

In this scenario, the advantage posed by the application of GMF to BMNP-cell interaction within the first minutes or, even, seconds after the treatment is worth noting and becomes crucial in increasing the effectivity of the treatment.

**Figure 3:**
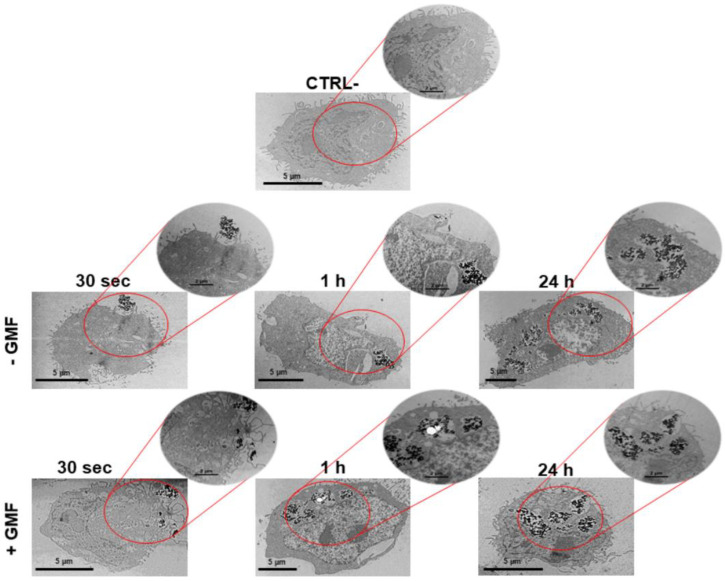
Interaction of BMNPs with 4T1 cells in the presence/absence of a continuous gradient magnetic field analyzed at TEM: Micrographs of the cells incubated with the 100 µg/mL of BMNPs for different periods of time. The micrographs are representative of alternate serial cuts of the cell pellets of each sample. Scale bar: 5 µm.

The interaction of BMNPs with cells was also analyzed at different time points by TEM that identified BMNPs through iron detection. In agreement with the data of optical microscopy after 30 s, only a few BMNPs were detected around the cell surface when cells were not subjected to the magnetic field. On the other hand, some BMNPs appeared to interact with the cell membrane and even to be internalized when a magnet was applied to the cells (Figure 3). The presence of iron in these samples was confirmed by microanalysis performed by energy dispersive X-ray (TEM-EDX) (Figure S2).

As expected, no significant differences between the two treatments were detected for the longer incubation times of 1 and 24 h. In both cases, BMNP internalization increased with time. Thus, the data obtained with TEM analysis are in accordance with those observed with Prussian blue staining and iron quantification. Altogether, these data show that BMNPs are highly responsive to a GMF in vitro, which allows an earlier and faster cellular interaction (Figure 2) and uptake (Figure 3). The faster internalization of BMNPs treated with a GMF is in good agreement with data reported by other authors in different cell lines, which also present enlarged endosomes where MNPs were accumulated in high amounts, without affecting cell viability [49,50]. Other researchers reported that, depending on the presence/absence of the magnet, a clear difference in the uptake of the MNPs was detected for at least 90 min [45]. Thus, our results show that the interaction and accumulation of BMNPs is reached earlier when cells are exposed to an external magnetic field as the magnetic force increases the sedimentation of BMNPs onto the cellular surface.

### 2.3. The Apposition of a GMF Enhances the Uptake of DOXO Coupled to BMNPs

**Figure 4:**
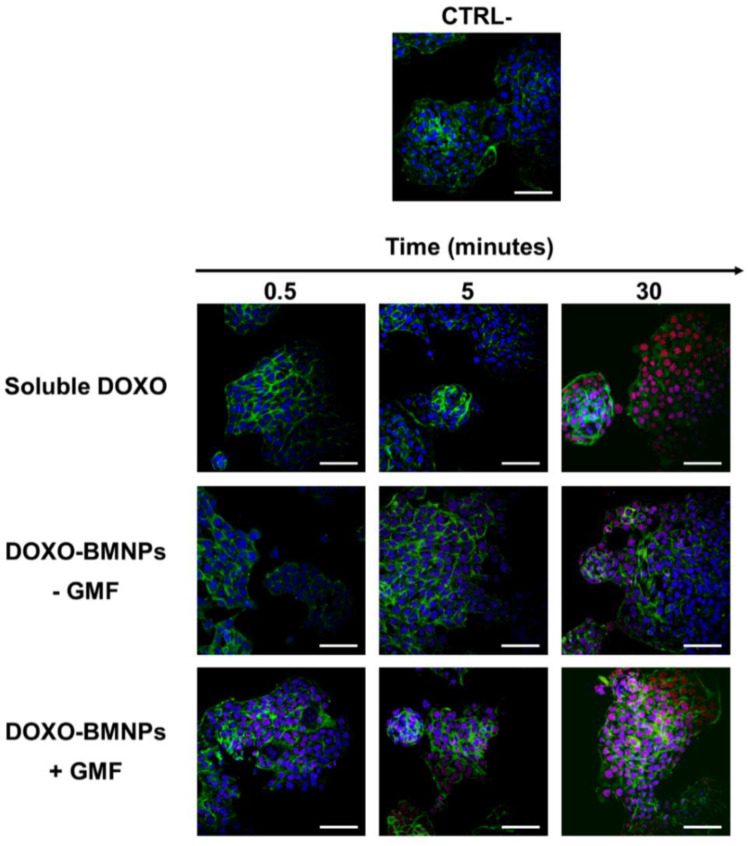
The apposition of a GMF enhances the cellular uptake of Doxorubicin (DOXO) coupled to BMNPs. 4T1 cells were incubated at 37 °C with DOXO-BMNPs for different times (0.5, 5, and 30 min) in the absence (−GMF) and presence (+GMF) of a gradient magnetic field. Soluble DOXO was used as a positive control. Cells were fixed, permeabilized, stained for cytoskeletal actin with fluorescein isothiocyanate (FITC)-phalloidin (green) and for nuclei with TO-PRO3 (blue) and visualized at confocal microscopy. DOXO was detectable for its intrinsic fluorescence in red. Scale bar: 50 µm.

Since BMNPs were planned as a drug delivery system, they were functionalized with DOXO and then incubated with 4T1 cells in the presence/absence of GMF. Their ability to deliver the drug to 4T1 cells was evaluated in experiments of confocal microscopy. DOXO, which was visualized in red by its intrinsic fluorescence, was already detectable in the cell nuclei after incubating functionalized BMNPs (100 ug/mL) in the presence of a magnet for 30 s, and the red signal increased over time (Figure 4).

By contrast, in the absence of the magnet, DOXO was observed within nuclei only after 30 min of incubation and, in any case, the signal was fainter. When soluble DOXO was incubated with cells, a strong red signal was detected but only after 30 min of incubation. The latter experiments are in line with others previously reported which showed that the cellular uptake of DOXO loaded on DOXO-BMNP nano-assemblies by cells was not as efficient as that of soluble DOXO [15,48]. This finding could probably be ascribed to the fact that soluble DOXO can easily diffuse through the plasma membrane, while DOXO associated with BMNPs either is internalized by a phagocytic pathway requiring a longer time or must first be released from the nanoparticles and then internalized. On the other side, the apposition of a magnet on DOXO functionalized BMNPs enhances cellular uptake of the drug when compared to freely diffusible soluble DOXO. Thus, the GMF favors quick concentration and accumulation of the drug in close contact with the cells and within the cells. Our results clearly show that DOXO adsorbed onto the BMNPs did not interfere with the applied magnetic field, in agreement with the data previously obtained by superconducting quantum interference device (SQUID) analysis [15].

### 2.4. The Apposition of a GMF Enhances the Cytotoxicity of DOXO-Coupled BMNPs

From the above experiments, it is clear that the application of a GMF enhances the interaction of BMNPs and its payload with cells when they are incubated for short times, but for longer periods BMNPs can interact even in the absence of GMF (Figure 2, Figure 3 and Figure 4). Indeed, in preliminary experiments in which cytotoxicity of DOXO-BMNPs at different concentrations was evaluated in MTT assays carried out for 72 h, either in the presence or in the absence of a GMF, no differences could be appreciated between the two treatments. This suggests that relatively long incubations do not allow for perceiving the difference.

**Figure 5:**
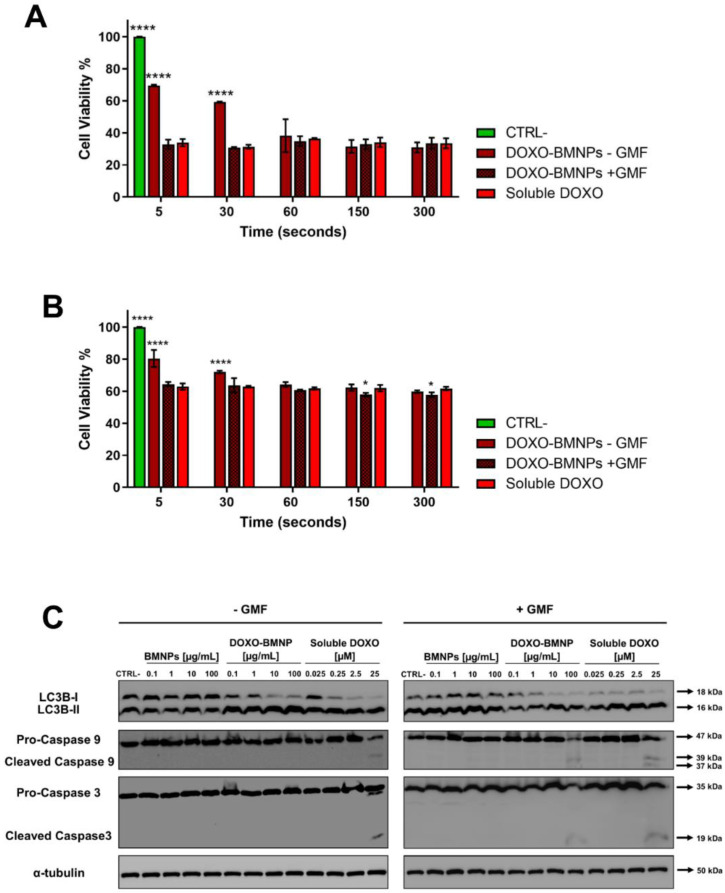
4T1 (**A**) and MCF-7 (**B**) cells were incubated with drug-loaded BMNPs and treated with a GMF for different times, after which BMNPs and media were withdrawn and cells were replenished with fresh media and incubated for a further 72 h in an MTT assay. Soluble DOXO was used as a positive control. In all experiments, untreated cells receiving medium without nanoparticles were taken as the reference value (100%) of viable cells. Data are the average of 3 independent experiments performed in triplicates. Differences between groups were assessed by 2-way ANOVA with Dunnett’s multiple comparison test (**** *p* < 0.0001). (**C**) Expression and state of activation of LC3b-I, caspase 3, and caspase 9. Extracts from 4T1 cells incubated for 16 h with different concentrations of BMNP ± DOXO in the absence/presence of a GMF were analyzed in Western blot. All blots shown are representative of three independent experiments. A Western blot representative of the bands of tubulin is shown, since in all experiments, similar patterns were observed.

For this reason, to evaluate potential differences between the cytotoxicity in the samples treated with the magnetic field or not, a kind of pulse-chase MTT assay was carried out. 4T1 and MCF-7 cells incubated with DOXO-BMNPs (100 µg/mL) underwent GMF treatment or not for short times (from 5 to 300 s), and then, the medium and the BMNPs were withdrawn and incubation with fresh media at 37 °C was continued for further 72 h before reading the test. In the presence of the magnetic field at the time points of 5 and 30 s, DOXO-BMNPs exerted the same level of cytotoxicity exerted by soluble DOXO, while in the absence of the magnetic field, DOXO-BMNPs exerted lower toxicity (Figure 5A,B).

This was observed for both cell lines, although MCF-7 showed a higher level of viability, possibly because these cells are more resistant to the effect of this drug [51]. It is expected that the application of the magnet in vivo at the tumor site could thus favor retention and accumulation of DOXO-BMNPs, which circulate in the bloodstream, promoting a higher toxicity against tumor cells [52,53]. Several scenarios could be envisaged regarding the interaction of DOXO-BMNPs with cells. Particles (or also some of them) could be uptaken by cells with their drug payload, or not internalized particles could release DOXO outside the cells and then the drug could be internalized. In any case, the application of a magnetic field had a strong effect in terms of DOXO internalization and induced cytotoxicity. Indeed, without apposition of the GMF, the cytotoxic effect was delayed. Finally, these data suggest that DOXO is underestimated by confocal visualization, since its cytotoxic activity precedes in time its detection.

DOXO promotes cell death by various mechanisms, which are generally classified as caspase-independent and caspase-dependent; the former comprises autophagy, which is also called type II cell death [54]. Indeed, autophagy is an essential finely tuned housekeeping mechanism that enables cells to maintain homeostasis and normal functions by degrading and recycling injured organelles and misfolded proteins [55], and its dysregulation in both directions in cancer cells can lead to cell death and its modulation can thus represent a therapeutic strategy [56]. We thus evaluated whether DOXO-BMNPs in absence/presence of a GMF were able to activate an autophagic pathway with respect to not functionalized BMNPs and to soluble DOXO, for which the maximum level of cell death is already detectable after 16 h [57]. Figure 5C and Figure S3A,B show that the incubation of 4T1 cells with the two highest concentrations (10 and 100 μg/mL) of DOXO-BMNPs and with the corresponding doses of soluble DOXO induced activation of LC3b-I, which was nearly all cleaved to LC3b-II, as detected in Western blot analysis. This activation of autophagy is strictly dependent on the presence of DOXO, since neither nonfunctionalized BMNPs had an effect nor the apposition of a GMF enhanced the DOXO-induced cleavage of LC3b-I, a well-recognized autophagy biomarker [58]. It is thus concluded that DOXO, either soluble or coupled to BMNPs, exerts cytotoxicity, while the cytocompatibility of BMNPs is confirmed also with this parameter.

Apoptosis is another arm of the mechanisms contributing to efficient antitumor action for most anticancer drugs, including DOXO [59,60], and the activation of intracellular caspases is one of the main characteristics of the apoptotic cell death pathway. Two caspases, in particular caspase 9, which is involved in the first apoptotic events as a initiator, and caspase 3, which is an executer [61,62], were thus analyzed in Western blot prepared from lysates obtained from cells undergoing the same treatments as above. As shown in Figure 5C and Figure S3, only if cells were treated with DOXO-BMNPs and GMF were the two caspases activated to their cleaved forms. In the controls, soluble DOXO could induce caspase cleavage independently of the apposition of the magnetic field, while not functionalized BMNPs had no effect both in the presence and absence of a GMF.

### 2.5. In Vivo Biocompatibility and Nanoparticles Biodistribution

After showing that BMNPs were cytocompatible in different in vitro assays, we analyzed the in vivo biocompatibility as well as their distribution in different organs after systemic administration by tail vein injection. We chose the dose of 10 µg BMNPs/g mouse, corresponding to about 8 µg of Fe/g mouse, according to studies previously published on Fe_3_O_4_ MNPs [63,64]. All mice injected with BMNPs were found to be alive and in good shape for at least 60 days, the latest time point checked. Sections of brain, heart, lung, spleen, liver, and kidney prepared from animals 1, 7, and 60 days after BMNP injection do not show any morphological alterations compared to those from a control mouse (Figure S4). In the case of spleen, while the specimens from untreated control animals were positive for Prussian blue staining because of their endogenous iron deposits, such a staining was undetectable 1 day after BMNP injection, but it was resumed 1 week after, if not earlier. Moreover, BMNPs are not retained in the different organs, except for a low amount in the lungs and in the liver. Other authors also detected magnetic nanoparticles in spleen, liver, and lungs [65,66] after their intravenous administration due to the vascularized nature of these organs but without associated toxicity. Moreover, these organs are part of the reticuloendothelial system (RES), where many different nanoparticles of the BMNP size range accumulates because they are phagocytosed by macrophages through adsorption of opsonin [63,67]. On the other side, it is widely accepted that iron present in the injected MNPs can be recycled in ferritin proteins [68] or eliminated with the feces [69].

Altogether, these data confirm the full biocompatibility of BMNPs up to 10 µg/g mouse weight, in agreement with the in vitro data and the previously reported results [14,15]. In fact, Kim et al. [70] demonstrated that doses of up to 100 mg/kg of MNPs, within the range of 50 nm, are not toxic after circulation for a month.

### 2.6. The Apposition of GMF Enhances the Antitumor Effect of DOXO-Coupled BMNPs

The antitumor efficacy of DOXO-BMNPs in combination with application of a GMF was studied in vivo in BALB/c mice bearing mammary carcinomas induced by intra fat pad mammary gland injection of 4T1 cells. This tumor model was chosen at the beginning of this study, since its growth and metastatic spread mimic very closely stage IV human breast cancer [71].

**Figure 6:**
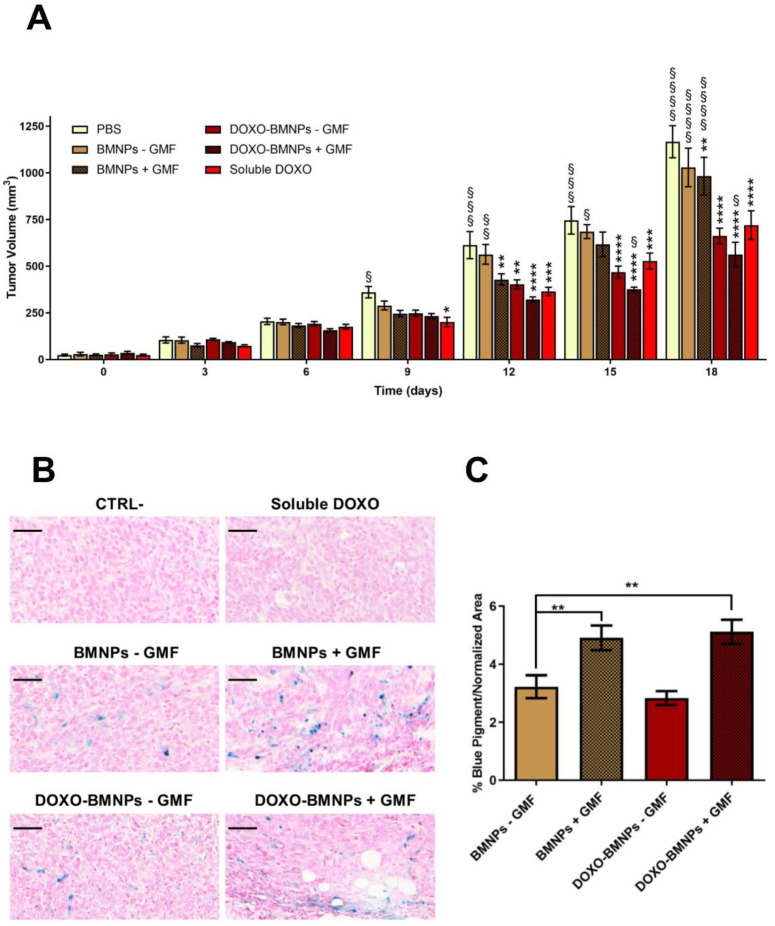
The apposition of GMF enhances the antitumor effect of DOXO-coupled BMNPs. (**A**) DOXO-BMNPs or BMNPs were injected intravenously in female BALB/c mice (*n* = 9) bearing tumors induced by 4T1, combined with GMF apposition or not. Each treatment was given 6 times, every 3 days starting from day 0, at a dose of 2 mg DOXO/kg mouse body weight or comparable amounts of BMNPs (15 µg BMNPs/g body weight). Controls included same amounts of soluble DOXO and PBS alone. Tumor sizes were measured every 3 days. The results are expressed as mean ± SD. Differences between groups were assessed by 2-way ANOVA with Dunnett’s multiple comparison test (**** *p* < 0.0001; *** *p* = 0.0001; ** *p* = 0.001; and * *p* = 0.01; * indicates samples compared to PBS) (§§§§ *p* < 0.0001; §§§ *p* = 0.0001; §§ *p* = 0.001; and § *p* = 0.01; § indicates samples compared to soluble DOXO). (**B**) Presence of BMNPs, detected by Prussian blue staining, and (**C**) iron quantification in histologic sections of the tumors at the end of the experiment (d 18). Scale bar: 50 µm. The results are expressed as mean ± SD. Differences between groups were assessed by 1-way ANOVA with Dunnett’s multiple comparison test (** *p* < 0.05).

Mice bearing tumors of approximately 30 mm^3^ were intravenously injected with BMNPs, DOXO-BMNPs, and soluble DOXO at the same corresponding amounts (2 mg/kg mouse) and with Phosphate Buffered Saline (PBS) as controls. Immediately after nanoparticle injection, in half of the mice, a GMF was applied on the tumor for 1 h. All treatments were repeated 5 more times at 3-day intervals and, each time, tumor sizes were evaluated and compared to the ones of control animals receiving only PBS or soluble DOXO. No differences between the groups receiving the different treatments were observed up to day 6 (Figure 6). At day 9, all treated animals displayed tumors with decreased sizes with respect to the controls receiving PBS.

From this moment on, significant differences emerged between the mice injected with DOXO versus those mice not receiving DOXO but injected with BMNPs ± GMF, with these differences being even more evident at the end of the experiment (day 18) when mice were euthanized for ethical reasons. The highest percentage of inhibition was observed in mice receiving the combined treatment of DOXO-BMNPs and apposition of GMF (52 ± 5%), versus animals receiving only DOXO-BMNPs (43 ± 3%) or soluble DOXO (38 ± 2%).

At the end of the experiment, tumors were excised and fixed and histologic sections were stained with Prussian blue to analyze and quantify the iron content. As expected, tumor sections from animals that were injected with BMNPs, both with the DOXO payload and without, and underwent GMF treatments displayed a higher level of the blue pigment revealing iron, which was quantified as being approximately double the amounts present in the tumors not treated with GMF. These values were similar in the two cases of BMNPs and DOXO-BMNPs (Figure 6B,C). This finding is in agreement with reports from other laboratories, which showed that the intravenously injected with synthetic MNPs accumulate from 2× to 8× within the target site when a GMF is applied there [72]. The presence of BMNPs in the tumors, even at low levels, in the absence of GMF treatment could be due to their passive accumulation related to the enhanced permeability and retention (EPR) effect. Indeed, for subcutaneous tumors with low vascularization, the EPR effect was reported to cause accumulation of about 1–15% of nanoparticles, relative to the injected dose, which was doubled upon apposition of the magnet doubled [72,73].

As a summary, our results confirm that DOXO-BMNPs maintain their ability to respond to a GMF also in vivo and can be directed to a specific organ/tumor for drug delivery or hyperthermia treatment (once arrived at the tumor site).

This targeted treatment potentially reduces the side effects of the drug on healthy cells in the rest of the body, thus favoring accumulation at the tumor site of the nanoparticles loaded with DOXO, which could exert its toxic effect. In this context, much work has been devoted to producing magnetically targeted chemotherapy treatments for tumors from different organs, including lung, prostate, brain, melanoma, breast, and liver, with the goal of achieving a high concentration of drugs in the affected area with a rapid response time and minimal side effects [74,75,76].

### 2.7. In Vitro Cytotoxicity of BMNPs under the Influence of An AMF

Finally, a step forward is to use the same nanoplatform as nanocarrier and as a magnetic hyperthermia agent to enhance the cytotoxic effect of chemotherapy with hyperthermia. Indeed, it is well known that magnetic nanoparticles exposed to an AMF develop heat, thus selectively killing tumor cells which are more sensitive than normal cells to high temperatures in the range of 43–46 °C [3,77]. Such a potential application was first tested in in vitro experiments. Briefly, 4T1 cells resuspended in Eppendorf tubes with different amounts of BMNPs were positioned under an AMF of 130 kHz and 18 kA m^−1^ for 20 min and cell viability was then analyzed in an MTT assay, which was read out 24 h after plating the cells. Cells were fully viable when incubated with 100 µg of BMNPs, both in the presence or absence of the AMF, and only by increasing BMNPs concentration was an effect of the applied AMF detected in a dose-dependent relationship (Figure S5). Indeed, when cells were incubated with 500 µg of BMNPs in the presence of the AMF, a temperature of about 45 °C was reached and cell viability was reduced to only 8.4%, while in the absence of the AMF, cell viability was still around 60% of the controls without BMNPs. It is thus clear from these data that the amount of BMNPs is critical to generate enough heating, which is then responsible for cell death [78].

### 2.8. AMF Enhances the in Vivo Antitumor Activity of DOXO-BMNPs

Since in vitro experiments showed that BMNPs could be used as hyperthermia agents, experiments were designed to demonstrate the effectiveness of the MH and the combination of this treatment with chemotherapy in vivo.

**Figure 7:**
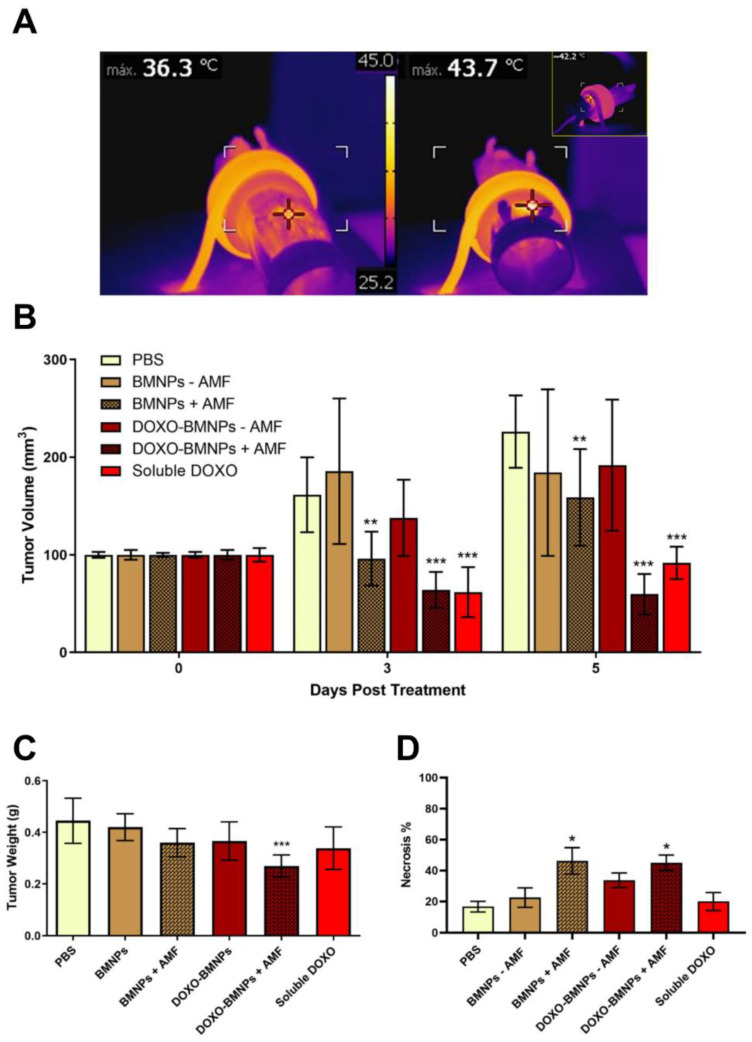
In vivo antitumor activity of BMNPs under the influence of an alternating magnetic field (AMF): (**A**) Images taken with a thermic camera of a representative mouse without (left) and with (right) injected BMNPs during AMF treatment. Note the different colors within the circle on the backside of the mouse. (**B**) Effect on the growth of 4T1 tumors (*n* = 8) in female BALB/c mice, analyzed 3 and 5 days after one single injection of DOXO-BMNPs or not functionalized BMNPs ± AMF: Each group received one intratumor injection of 3 mg BMNPs/mouse on the first day of the treatment (day 0). For the groups injected with soluble DOXO or DOXO-BMNPs, the dose of DOXO (either soluble or adsorbed on the BMNPs) was 80 µg/mouse. Differences between groups (all compared to PBS) were assessed by 2-way ANOVA with Dunnett’s multiple comparison test (*** *p* < 0.0001; ** *p* = 0.001). Weight (**C**) and necrosis % (**D**) of tumors were measured at the end of the experiment (day 5), and all samples were compared to the PBS group. Differences between groups were assessed by ordinary one-way ANOVA with Dunnett’s multiple comparison test (*** *p* < 0.05; * *p* = 0.001).

Mice bearing 4T1-induced tumors were injected once in situ with DOXO-BMNPs and DOXO-free BMNPs, and then half of the mice in each group were subjected to AMF. Controls included mice injected with the same doses of soluble DOXO (positive control) or PBS (negative control). When an AMF was applied in vivo, hyperthermia production was observed only in the mice injected with the NPs (Figure 7A, right vs left panel). Tumor temperature was found to reach 42–45 °C in the first 2–3 min, and this temperature was maintained throughout the treatment lasting 20 min.

At day 3 posttreatment, the best therapeutic effects were observed in mice treated either with soluble DOXO or with DOXO-BMNPs and were subjected to AMF (Figure 7B). In both cases, tumor volumes were significantly reduced, while in all the other cases, tumors increased their sizes, except for mice injected with DOXO-free BMNPs and subjected to AMF, where tumor volumes were stable.

At day 5 postinjection, the treatment with the strongest therapeutic benefit was DOXO-BMNPs + AMF. Mice from this specific group displayed tumor volumes virtually identical to those registered at day 3 posttreatment, while those from mice treated with soluble DOXO and BMNPs + AMF showed larger volumes compared to those measured at day 3 posttreatment, but still, these two treatments displayed better therapeutic effect compared to all the other treatments. A first conclusion from these experiments is that AMF-induced hyperthermia is a valid treatment to reduce tumor size. Regarding tumor weight, at day 5 posttreatment, this parameter was significantly reduced only in tumors from mice treated with DOXO-BMNPs + AMF (Figure 7C). Also, significantly higher necrosis of the cancer cells was observed in tumors from mice treated with NPs +AMF, irrespective of the presence or absence of DOXO based on results of Hematoxylin & Eosin staining of tumor tissue (Figure 7D). Indeed, in these cases, necrotic areas were around 45–46% of the tumor area, compared to those observed in all the other experimental conditions (20% of the tumor area). Combining the results of the two latter parameters, it is thus clear that DOXO-BMNPs together with hyperthermia have a strong antitumor efficacy.

In conclusion, DOXO coupled to BMNPs has a longer lasting and more efficient effect, possibly because, as a nano-assembly, it remains in the tumor site for longer periods, with the drug being slowly released following changes in the environmental pH values and also triggered by hyperthermia, as previously demonstrated by Peigneux et al. [15]. Therefore, higher DOXO doses are locally reached at the target site that, along with the local temperature increase triggered by application of the AMF, allow for better efficiency of the treatment. Although still not ideal, BMNPs have improved colloidal stability compared to MNPs, even if they are larger than the latter. Ways to improve colloidal stability that are presently investigated by our research group are embedding the BMNPs in liposomes [34], covering the BMNPs with protein corona from plasma [38], and/or mixing BMNPs and MNPs [39].

## 3. Materials and Methods

### 3.1. BMNP Synthesis

MamC protein was heterologous expressed in *E. coli* TOP10 competent (Life Technologies: Invitrogen, Grand Island, NY, USA), purified by affinity chromatography under denaturing conditions (IMAC, GE Healthcare, Chicago, IL, USA), and refolded by serial dialysis steps as previously described by Valverde-Tercedor et al. [32]. Then, the biomimetic magnetic nanoparticles (BMNPs) were synthesized inside an anaerobic Coy chamber (96% N_2_/4% H_2,_ Coy Laboratory Products, Grass Lake, MI, USA) at 25 °C and 1 atm total pressure following the protocol described by Peigneux et al. [15]. Briefly, 10 µg/mL of MamC protein was added to deoxygenated solutions of 2.78 mM Fe(ClO_4_)_2_, 5.56 mM FeCl_3_, and 3.5 mM/3.5 mM NaHCO_3_/Na_2_CO_3_ for the in vitro coprecipitation reaction. BMNPs were incubated for 30 days and then were washed three times with deoxygenated Milli-Q water, each time by concentrating BMNPs in the vial with a N42 neodymium magnet one (1.8 kg pull, Magnet Expert Ltd.; 10 mm diameter × 3 mm thickness) placed outside the vial, discarding the fluid, adding fresh water, shaking vigorously, and discarding the fluid again. BMNPs were kept in water inside the Coy chamber until further use. The concentration of BMNPs in suspension was calculated by weight difference taken in a precision scale between a given volume of a BMNP suspension and the same sample once all water was evaporated by using a thermoblock at 100 °C. This concentration was measured independently in, at least, five different samples from the same batch, and an average concentration value was taken. 

### 3.2. Functionalization of the BMNPs Produced in Presence of MamC Protein

BMNPs were functionalized with doxorubicin (DOXO-BMNPs) following the same procedure carried out previously [15]. Briefly, 5 mg of BMNPs was mixed with 1 mg/mL of DOXO dissolved in water inside hermetic closed bottles to avoid magnetite oxidation. Mixtures were maintained at 25 °C in rotation on a wheel for 24 h. Then, the DOXO content was assessed by UV-Vis spectroscopy (λ = 490) with Nanodrop, indicative of the concentration of the molecule by comparison to a standard curve. The amount of adsorbed DOXO was calculated from the differences between the concentration of the molecule in the supernatant before and after adsorption on the BMNPs. The solid components were washed 5 times with 4-(2-Hydroxyethyl)piperazine-1-ethanesulfonic acid, N-(2-Hydroxyethyl)piperazine-N′-(2-ethanesulfonic acid) buffered saline solution (0.01 M HEPES and 0.15 M NaCl) until the absorbance was less than 0.02 units at 490 nm (equivalent to a negligible amount) using a magnet. Each washing was performed by concentrating BMNPs in the vial with the neodymium magnet placed outside the vial, discarding the fluid, adding fresh HEPES buffered saline solution, shaking vigorously, and discarding the fluid again. Then, the functionalized nanoparticles were resuspended in the same solution and kept at 4 °C until further use. The concentration of functionalized BMNPs was measured by iron quantification with potassium thiocyanate. Both BMNPs and functionalized BMNPs were dissolved in 37% HCl, mixed with 10% H_2_O_2_, and incubated for 20 min at room temperature. Samples were then stained with 1 mL of 1% potassium thiocyanate in Milli-Q water, and their absorbances were measured at 490 nm. The concentration of ferric ions in the samples was calculated referencing the obtained absorbances to a standard curve performed following the same protocol with known concentrations of BMNPs.

### 3.3. Cell Cultures

The 4T1 murine breast carcinoma cell line derived from BALB/c mice (ATCC^®^ CRL-2539™) were maintained in Dulbecco’s Modified Eagle Medium (DMEM) supplemented with 10% fetal calf serum (FCS), 50 U/mL penicillin, and 50 μg streptomycin (here referred as complete medium). Cells were sub-cultured twice a week, when they were at 80–90% confluence.

### 3.4. Interactions of BMNPs with Cells in the Absence/Presence of a GMF

#### 3.4.1. Detection of Reactive Oxygen Species (ROS) Production

To measure the potential oxidative stress in living cells, as a consequence of the presence of the BMNPs, the CellROX^®^ Green Reagent (ThermoFisher, Waltham, MA, USA) was used following the protocol recommended by the manufacturer. Briefly, cells (approximately 20 × 10^3^ 4T1/well) were seeded on glass coverslips in 24-well plates. After exposure to different concentration of BMNPs (0.1, 1, 10, and 100 μg/mL) in the presence and absence of a gradient magnetic field for 4 h, the cells were washed with PBS and CellROX^®^ Green Reagent was added to a final concentration of 5 μM in 300 µL of DMEM medium without serum. Then, the plate was incubated in the dark at 37 °C for 30 min. Menadione (100 µM) was used as a positive control [40]. After the incubation time, the coverslips were washed with PBS pH 7.2, fixed with 4% paraformaldehyde in PBS, washed again, and permeabilized with 0.1% Triton-X100 for 10 min. Finally, the coverslips were stained and mounted on specimen slides (Biosigma). The cytoskeletal actin was stained with TRITC-phalloidine (1/200, Sigma-Aldrich (St. Louis, MO, USA), excitation at 543 nm; emission at 560–620 nm), and the cell nuclei were stained with TO-PRO-3 (1/50, Life Technologies, excitation at 642 nm, emission at 650–750 nm). The CellROX^®^ Green Reagent is only fluorescent in the oxidized state because of ROS production. Therefore, the emission of green fluorescence (at 485/520 nm) is stable and is produced after DNA binding, and therefore, its signal is mainly located in the nucleus. Fluorescence was detected using a Spectral Confocal Leica TCS SP2 AOBS microscope. The images were taken at 400× magnification. The ImageJ software was used for the analysis.

#### 3.4.2. MTT Assay in the Absence/Presence of a GMF or an AMF

Cells (approximately 5 × 10^3^ 4T1/well) were incubated in 96-well plates for 24 h. Then, different concentrations of BMNPs (0.1, 1, 10, and 100 µg/mL) were added to plated cells in 100 μL of complete medium. These samples were incubated at 37 °C and 5% CO_2_ in the absence or presence of a gradient magnetic field, using a magnetic plate below the 96-well plates, for 72 h. In another set of experiments, the cells were incubated with 100 µg/mL of BMNPs, DOXO-BMNPs, and a quantity of soluble DOXO normalized for the amount of drug adsorbed on BMNPs for shorter time points (5, 30, 60, 150, and 300 s), both in the presence and absence of the gradient magnetic field.

In the case of the alternating magnetic field treatment, approximately 95 × 10^4^ 4T1 cells were placed in a 0.5 mL tube. Then, suspensions of 100, 300, and 500 µg of BMNPs, resuspended in complete DMEM medium, were added and exposed or not to an alternating magnetic field (130 kHz and 18 kA m^−1^) for 20 min. After this treatment, cells were counted by using trypan blue, seeded in 96-well plates (approximately 10 × 10^3^ 4T1/well), and incubated at 37 °C and 5% CO_2_ for 24 h.

At the end of the incubation time of the different experiments, cell viability was evaluated by MTT colorimetric assay as described in Oltolina et al. [46]. Briefly, 20 μL of MTT solution (5 mg/mL in PBS solution) was added to each well. The plate was then incubated at 37 °C for 2 h, and then, supernatants were carefully aspirated. Afterwards, 100 μL of 0.2 N HCl in isopropanol was added to dissolve the formazan crystals formed, and the optical density was measured in a multiwell reader (2030 Multilabel Reader Victor TM X4, PerkinElmer, Waltham, MA, USA) at 570 nm. Viability of untreated cells was taken as 100% viability, and values obtained from cells undergoing the different treatments were referred to this value. Experiments were performed at least for 3 times using 3 replicates for each sample.

#### 3.4.3. Prussian Blue Staining

Cells (approximately 20 × 10^3^ 4T1/well) were seeded on glass coverslips in 24-well plates, and after 24 h, 100-µg/mL BMNP suspensions were added. After the incubation at 37 °C for short (5 and 30 s) and longer periods of time (1, 2.5, and 5 min) in the absence and the presence of a GMF, coverslips were washed with fresh PBS pH 7.2 and fixed with paraformaldehyde (2 wt% in PBS). Then, Prussian blue solution (1:1 of 2% potassium ferrocyanide in H_2_O and 2% HCl both in H_2_O) was added to the coverslips. In that way, any ferric ion (+3) present in the samples combines with the ferrocyanide and results in the formation of bright blue pigments called Prussian blue or ferric ferrocyanide. After two other washes with fresh PBS, Nuclear Fast Red (Sigma-Aldrich) was added for staining cell nuclei. Finally, coverslips were washed with H_2_O and mounted on slides by using one drop of Eukitt quick-hardening mounting medium for each sample. The interaction of the stained BMNPs with cells was analyzed by optical microscopy at 100×. Experiments were performed at least 3 times.

#### 3.4.4. Iron Quantification by Potassium Thiocyanate

Cells (approximately 22 × 10^4^ 4T1/well) were seeded in 6-well plates and, after 24 h incubation at 37 °C and 5% CO_2_, 100-µg/mL BMNP suspensions in complete DMEM medium were added. After their incubation for 5, 30, 60, 150, and 300 s in the presence and absence of a GMF, the supernatant was removed, and cells were washed with fresh PBS, trypsinized, transferred to 0.5 mL tubes, and centrifuged at 1000 rpm for 5 min. Then, the cell pellets formed were dissolved in 37% HCl, mixed with 10% H_2_O_2_, and incubated for 20 min at room temperature. After the incubation time, the samples were reacted with 1 mL of 1% potassium thiocyanate in Milli-Q water, and their absorbance was measured at 490 nm. The concentration of ferric ions in the samples was calculated in reference to the absorbance obtained from a standard curve performed following the same protocol as that with the BMNPs alone. The endogenous iron of cells was subtracted from the treated samples normalized by the untreated control cells. Experiments were performed at least 3 times.

### 3.5. Internalization of BMNPs and DOXO in CELLs

#### 3.5.1. Cellular Internalization by TEM

Cells (approximately 10 × 10^5^ 4T1/well) were incubated at 37 °C and 5% CO_2_ for 24 h. Afterwards, 100 µg/mL of BMNPs were added and were incubated in the absence and presence a magnetic gradient field for 30 s and for 1 and 24 h. After these treatments, cells were washed three times with PBS prior to fixation with 2.5% glutaraldehyde and 2% paraformaldehyde in PBS for 1 h. Then, samples were washed again three times with sodium cacodylate buffer and embedded in Epon. Ultrathin sections (50–70 nm) were cut using a Reichert Ultracut S microtome (Leica Microsystems GmbH, Wetzlar, Germany), mounted on copper grids, and stained with lead citrate and uranyl acetate for transmission electron microscopy (TEM) analysis.

#### 3.5.2. DOXO Internalization Analysis

Cells (approximately 20 × 10^3^ 4T1/well) were seeded on glass coverslips in 24-well plates and, after 24 h, 100 ug/mL of DOXO-BMNP suspensions or an amount of soluble DOXO (as a positive control) normalized for the one loaded on BMNPs was added. After incubation at 37 °C for different periods of time (30 s and 5 and 30 min) in the absence (−GMF) and the presence (+GMF) of a gradient magnetic field, coverslips were washed with fresh PBS pH 7.2 and fixed with paraformaldehyde (2 wt% in PBS). To minimize unspecific interactions and permeabilize cells, coverslips were washed with Tris-Buffered Saline (TBS) containing 5% Bovine Serum Albumin (BSA), 0.1% Triton X-100, and 5% goat serum and were then stained. In particular, cytoskeletal actin microfilaments were stained with FITC-phalloidin (Sigma-Aldrich, excitation at 488 nm; emission at 500–535 nm) and nuclei with TO-PRO-3 (1/70, Life Technologies; excitation at 633 nm; emission at 650–750 nm). DOXO was detected after excitation at 476 nm and emission at 575–630 nm. Fluorescence was detected using a Leica TCS SP2 AOBS Spectral Confocal Scanner microscope. Images were taken at 400× magnification. ImageJ software was used for analysis.

### 3.6. Western Blot Analysis

4T1 cells (approximately 22 × 10^4^ 4T1/well) were seeded in 6-well plates and, after 24 h incubation at 37 °C and 5% CO_2_, were treated for 16 h with different concentrations of BMNPs, DOXO-BMNPs (0.1, 1, 10, and 100 μg/mL), and an amount of soluble DOXO normalized for the one adsorbed to BMNPs (0.025, 0.25, 2.5, and 25 μΜ) in the presence or absence of a gradient magnetic field. Cells were then washed twice in cold PBS and lysed in iced Radioimmunoprecipitation assay buffer (RIPA) buffer (20 mM Tris-HCl pH 7.5, 150 mM NaCl, 50 mM HEPES, 0.1% SDS, 1 mM Ethylene glycol-bis(2-aminoethylether)-N,N,N′ (EGTA), 1% NP-40, 1% sodium deoxycholate, 2.5 mM sodium pyrophosphate, and 10% glycerol) supplemented with protease inhibitors cocktail (Sigma-Aldrich). Cell lysates were centrifuged at 13,000 rpm at 4 °C for 15 min. Clarified cell extracts (30 µg of protein) were denatured by heating for 5 min at 95 °C in reducing Laemmli buffer; proteins were separated in an appropriate concentration of sodium dodecyl sulfate-polyacrylamide gel electrophoresis (SDS-PAGE) and transferred onto polyvinylidene difluoride (PVDF) filters. Filters were blocked with 5% non-fat dry milk for 2 h, rinsed in water, and probed with different antibodies in Tris-buffered saline (TBS), pH 8.0, 5% BSA, overnight at 4 °C. The list of primary antibodies used is reported below (Table 1). After extensive washing, immunocomplexes were detected with appropriate horseradish peroxidase-conjugated secondary anti-IgG antibodies (diluted 1/5000), followed by enhanced chemiluminescence (ECL kit; Biorad), and were analyzed in a Versadoc instrument (Bio-Rad Laboratories S.r.l, Segrate, Milan, Italy). The Image Lab™ Software (Bio-Rad Laboratories Inc., Hercules, CA, USA) was used to perform densitometric analysis of the Western blots. Experiments were performed at least 4 times. The uncropped Western blot can be found at Figure S6.

### 3.7. Magnetic Hyperthermia Measurement in Vitro and Vivo

Both in vitro and in vivo experiments were carried out using a homemade AC current generator consisting of a resonant LC circuit set at 130 kHz and a magnetic field strength of 18 kA/m (HF~2.34 × 10^9^ Am^−1^s^−1^). In any case, the product of field strength and frequency is within the safe limits of 4.85 × 10^8^ Am^−1^s^−1^ [79] or H-f ≤ 5 × 10^9^ Am^−1^s^−1^ proposed by Aktinson or later by Dutz and Hergt [80]. The magnetic field applicator consisted of a four-turn coil water-cooled copper pipe with 4-mm inside diameter. The temperature was monitored with a high-resolution infrared camera FLIR E60 with 320 × 240 pixel Infrared (IR) resolution and thermal sensitivity < 0.05 °C (FLIR Systems, Inc.) in real time. In all experiments, the temperature inside the coil was maintained at 37 °C. these values exceeded

### 3.8. In Vivo Test

#### 3.8.1. Animals

All Balb/c female mice of about six weeks old used in this work were purchased from Charles River (Calco, Lecco, Italy) and housed under standard conditions in a pathogen-free environment. All procedures were approved (Ministero della Salute: #178/2019-PR) and carried out in accordance with the Animal Care and Use Committee of UPO, the European Community Directive for Care and Italian Laws on animal experimentation (Law by Decree 116/92).

#### 3.8.2. In Vivo Magnetic Targeting and Antitumor Activity

Fifty-four female BALB/c mice were inoculated with 10^5^ 4T1 cells into the fat pad of mammary glands. When the tumors became palpable (10 days after cell inoculation), mice were divided into 6 different groups with comparable tumor volumes among the groups. The six groups of mice were intravenous injected and treated as follow: (i) PBS (negative control experiment), (ii–iii) BMNPs ± GMF, (iv–v) DOXO-BMNPs ± GMF, and (vi) soluble DOXO. Mice were injected 5 times with a dose of 2 mg/kg DOXO either soluble (positive control experiment) or as DOXO-BMNPs nano-assemblies (always maintaining the same DOXO concentration in any form) 3 days apart each time. In case of BMNP-bearing treatments, after each injection, the neodymium magnet was immediately attached with 3MTM Vetbond^TM^ tissue adhesive on the tumor site and kept for 1 h. This neodymium magnet, with a magnetic anisotropy perpendicular to the plane and a saturation magnetization of 800 emu/cc, has an effect equivalent to the application of a local direct current GMF of the order of 100 Oe a few millimeters from the tumor surface.

Throughout the study, tumor volumes (measured with a caliper) were recorded every 3 days. Three days after the last injections (day 18), mice were euthanized, and then, tumors, hearts, livers, spleens, brains, lungs, and kidneys were collected for histologic analysis. Histologic sections of the tumors were prepared for hematoxylin-eosin and Prussian blue staining to analyze particle biodistribution. The % of blue Prussian staining and standard area from 5 randomly chosen areas from each of the 3 tumor sections (100 microns apart) for each of the 7 tumors (*n* = 135) were analyzed by using ImageJ software.

#### 3.8.3. In Vivo Magnetic Hyperthermia and Antitumor Activity

Twenty-four female BALB/c mice were inoculated into the fat pads of two mammary glands with 10^5^ 4T1 cells each. Approximately 15 days after cell inoculation, when the tumor dimensions were approximately 100 mm^3^, mice were divided into 6 different groups with comparable tumor volumes among the groups. The 6 groups were intratumor injected and treated as follow: (i) PBS (negative control experiment), (ii–iii) BMNPs ± AMF, (iv–v) DOXO-BMNPs ± AMF, and (vi) soluble DOXO (positive control experiment). Mice were injected only once at the beginning of treatment (day 0) with a dose of 3 mg BMNPs/mouse, equivalent to 80 µg DOXO for the soluble DOXO and DOXO-BMNP groups. Immediately after injection of the nanoparticles, some groups were exposed to an AMF (130 kHz and 18 kA m^−1^) for 20 min. Throughout the study, tumor volumes were measured with a caliper every two days. Finally, five days posttreatment, mice were euthanized and their tumor weights recorded. Tumors were collected, fixed, embedded in paraffin, and processed for histologic analysis. Serial sections were stained with hematoxylin-eosin (Sigma Aldrich), and the percentage of necrosis was evaluated by a pathologist not informed of the sample identity.

### 3.9. Statistical Analysis

Data are expressed as mean ± standard error of at least 3 triplicates. Both for in vitro and in vivo test, statistical analyses were performed using a two-way ANOVA, with a Dunnet’s multiple comparisons test for grouped analyses using GraphPad Prism version 8.4.2 for Windows, GraphPad Software (GraphPad Prism, San Diego, CA, USA). Statistical differences between the treatments were considered significant when *p* values were *p* < 0.05 (*), *p* < 0.01 (**), *p* < 0.001 (***), and *p* < 0.0001 (****). Only for the in vivo experiments related to the weight of the tumor and for the percentage of necrosis, statistical analyses were performed using a one-way ANOVA, with a Dunnet’s multiple comparisons test for grouped analyses.

## 4. Conclusions

We performed preclinical studies aimed at validating biomimetic magnetic nanoparticles (BMNPs) as a drug (DOXO) delivery system, which can be manipulated externally by a gradient magnetic field (GMF) mediating tumor targeting or by an alternating magnetic field (AMF) developing hyperthermia in a mammary carcinoma model.

Results from the present study demonstrate that BMNPs are highly compatible both in vitro and in vivo. The apposition of a magnet (GMF) improves drug delivery and allows guidance of the nano-assembly to the tumor. In fact, our results show that GMF enhances the interaction of BMNPs with tumor cells and their toxicity if loaded with DOXO, both in vitro and in vivo. They also show that intravenously injected DOXO-BMNPs can be guided to the tumor mass by apposition of a magnet with a better therapeutic result than that produced by soluble DOXO.

Finally, our study shows that the combinatory chemothermal therapy mediated by BMNPs have a stronger therapeutic benefit compared to that carried out by soluble DOXO, possibly because BMNPs retain DOXO at the tumor site for longer periods and because the susceptibility of tumor cells to heat generated by hyperthermia can be simultaneously exploited by using the same BMNPs as a hyperthermia agent. These BMNPs are thus novel and promising nanocarriers for translational applications in cancer therapy.

## Figures and Tables

**Table 1:** Antibody used for Western blot analysis.

Antigen	Species	Dilution	Expected Band (kDa)	Source	Cat. Number
LC3B	Rabbit Polyclonal	1/500	16–18	Sigma-Aldrich	L7543
Caspase 9	Mouse Monoclonal	1/1000	37–39–47	Cell Signaling Technology	9508
Caspase 3	Rabbit Polyclonal	1/1000	19–35	Cell Signaling Technology	9662
α-tubulin	Mouse Monoclonal	1/500	50	Millipore	05-829

## Supplementary Materials

The following are available online at https://www.mdpi.com/2072-6694/12/9/2564/s1. Figure S1: Expression and state of phosphorylation of MAPK1/2, Akt, and mTOR, Figure S2: Microanalysis by energy dispersive X-ray (EDX) spectroscopy of 4T1 cells incubated with BMNPs, Figure S3: Densitometric analysis of the bands of activated LC3B-I, caspase 9, and caspase 3, Figure S4: Biodistribution profile of BMNPs after tail-vein injection in BALB/c mice, Figure S5: Cytocompatibility/cytotoxicity of different concentrations of BMNPs on 4T1 cells in the absence/presence of an alternating magnetic field (AMF) assessed as cell viability in an MTT assay. Figure S6: Uncropped Western blot.
