# Supplementary material for: Biomimetic Magnetite Nanoparticles as Targeted Drug Nanocarriers and Mediators of Hyperthermia in an Experimental Cancer Model
Source: Cancers (Basel). 2020 Sep 9;12(9):2564. doi: 10.3390/cancers12092564 (PMC7564965; doi:10.3390/cancers12092564)
Supplement: Supplementary file 1 [file cancers-12-02564-s001.pdf]

Supplementary Materials: Biomimetic Magnetite Nanoparticles as Targeted Drug Nanocarriers and Mediators of Hyperthermia in an Experimental Cancer Model

Francesca Oltolina, Ana Peigneux, Donato Colangelo, Nausicaa Clemente, Annarita D'Urso, Guido Valente, Guillermo R. Iglesias, Concepcion Jiménez-Lopez, Maria Prat

Cell cultures

The 4T1 murine breast carcinoma cell line derived from BALB/c mice (ATCC® CRL-2539™) were maintained in Dulbecco's Modified Eagle Medium (DMEM), supplemented with 10% fetal calf serum (FCS), 50 U/mL penicillin, and 50 µg streptomycin (here referred as complete medium). Cells were transplanted twice a week, when they were at 80-90% confluence.

EDX Analysis

Cells (approximately 10×10^5 4T1/well) were incubated at 37 ºC and 5% CO_2 for 24 h. Afterwards 100 µg/mL of BMNPs were added and were incubated in the absence and presence a magnetic gradient field for 30 seconds, 1 and 24 h. After these treatments, cells were washed three times with PBS prior to fixation with 2.5% glutaraldehyde and 2% paraformaldehyde in PBS for 1 h. Then samples were washed again three times with sodium cacodylate buffer and embedded in Epon. Ultrathin sections (50–70 nm) were cut using a Reichert Ultracut S microtome (Leica Microsystems GmbH, Wetzlar, Germany), mounted on copper grids, and stained with lead citrate and uranyl acetate for transmission electron microscopy (TEM) analysis. In addition, microanalysis by energy dispersive X-ray (EDX) spectroscopy was performed to confirm the BMNPs imagining by iron detection.

Western Blot Analysis

4T1 cells (approximately 22×10^4 4T1/well) were seeded in 6-well plates and, after 24 h incubation at 37 ºC and 5% CO_2, were treated for 16 hours with different concentrations of BMNPs, DOXO-BMNPs (0.1, 1, 10, 100 µg/mL) and an amount of soluble DOXO normalized for the one adsorbed to BMNPs (0.025, 0.25, 2.5, 25 µM) in presence or absence of a gradient magnetic field. Cells were then washed twice in cold PBS and lysed in iced RIPA buffer (20mM Tris-HCl pH 7.5, 150mM NaCl, 50mM HEPES, 0.1% SDS, 1mM EGTA, 1% NP-40, 1% sodium deoxycholate, 2.5mM sodium pyrophosphate, 10% glycerol) supplemented with protease inhibitors cocktail (Sigma-Aldrich). Cell lysates were centrifuged at 13,000 rpm at 4C° for 15 minutes. Clarified cell extracts (30 µg of protein) were denatured by heating for 5 minutes at 95C° in reducing Laemmli buffer, proteins were separated in an appropriate concentration of sodium dodecyl sulfate–polyacrylamide gel electrophoresis (SDS-PAGE) and transferred onto polyvinylidene difluoride (PVDF) filters. Filters were blocked with 5% non-fat dry milk for 2 hours, rinsed in water, and probed with different antibodies in Tris-buffered saline (TBS), pH 8.0, 5% BSA, overnight at 4°C. The list of the primary antibodies used is reported below. After extensive washing, immunocomplexes were detected with appropriate horseradish peroxidase-conjugated secondary anti-IgG antibodies (diluited 1/5000), followed by enhanced chemiluminescence (ECL kit; Biorad) and analyzed in a Versadoc instrument (*Bio-Rad* Laboratories S.r.l, Segrate, Milan, Italy). The Image Lab™ Software (Bio-Rad Laboratories Inc., Hercules, CA, USA) was used to perform the densitometric analysis of the Western blots. Experiments were performed at least 4 times.

Table 1. Antibody used for western blot analysis Table 1. Antibody used for western blot analysis.

Antigen	Species	Diluiton	Expected Band (kDa)	Source	Cat. Number
phospho-MAP Kinase 1/2 (Thr185/Tyr187)	Rabbit Monoclonal	1/1000	42 - 44	Millipore	04-797
MAP Kinase 1/2	Rabbit Polyclonal	1/700	42 - 44	Millipore	ABS44
phospho-Akt (Ser473)	Rabbit Polyclonal	1/500	60	Cell Signaling Technology	9271
Akt	Rabbit Polyclonal	1/500	60	Cell Signaling Technology	9272
phospho-mTor (Thr2446)	Rabbit Polyclonal	1/500	289	Millipore	09-345
mTor	Mouse Monoclonal	1/500	289	Millipore	05-1592
α-tubulin	Mouse Monoclonal	1/500	50	Millipore	05-829

In vivo biocompatibility and nanoparticles biodistribution

Twelve BALB/c mice were injected in the tail vein with 10 µg of BMNPs/g mouse weight diluted in a final volume of 100 µl of sterile PBS. Animals were monitored every two days up to 2 months. Mice were subdivided in 4 groups, differing for the time point of euthanization (from 1 day up to 2 months). Each group were composed by 4 mice. Their organs were collected, fixed, embedded in paraffin, and processed for histological analysis. Serial sections were stained with Prussian blue and Nuclear red (Sigma Aldrich) and then subjected to histological evaluation by an independent pathologist not informed of the sample identity.

In vitro cytotoxicity of BMNPs under the influence of an AMF

4T1 cells (10^6) were resuspended in a final volume of 0.15 mL in Eppendorf tubes in the presence of different amounts of BMNPs and positioned inside the coil under an AMF of 130 kHz and 18 kA m^{-1} for 20 min. Viability of the cells after the treatments was then analyzed in an MTT assay, which was read out 24h after plating the cells. The data were compared to cells incubated with the same amounts of BMNPs, but not subjected to AMF.

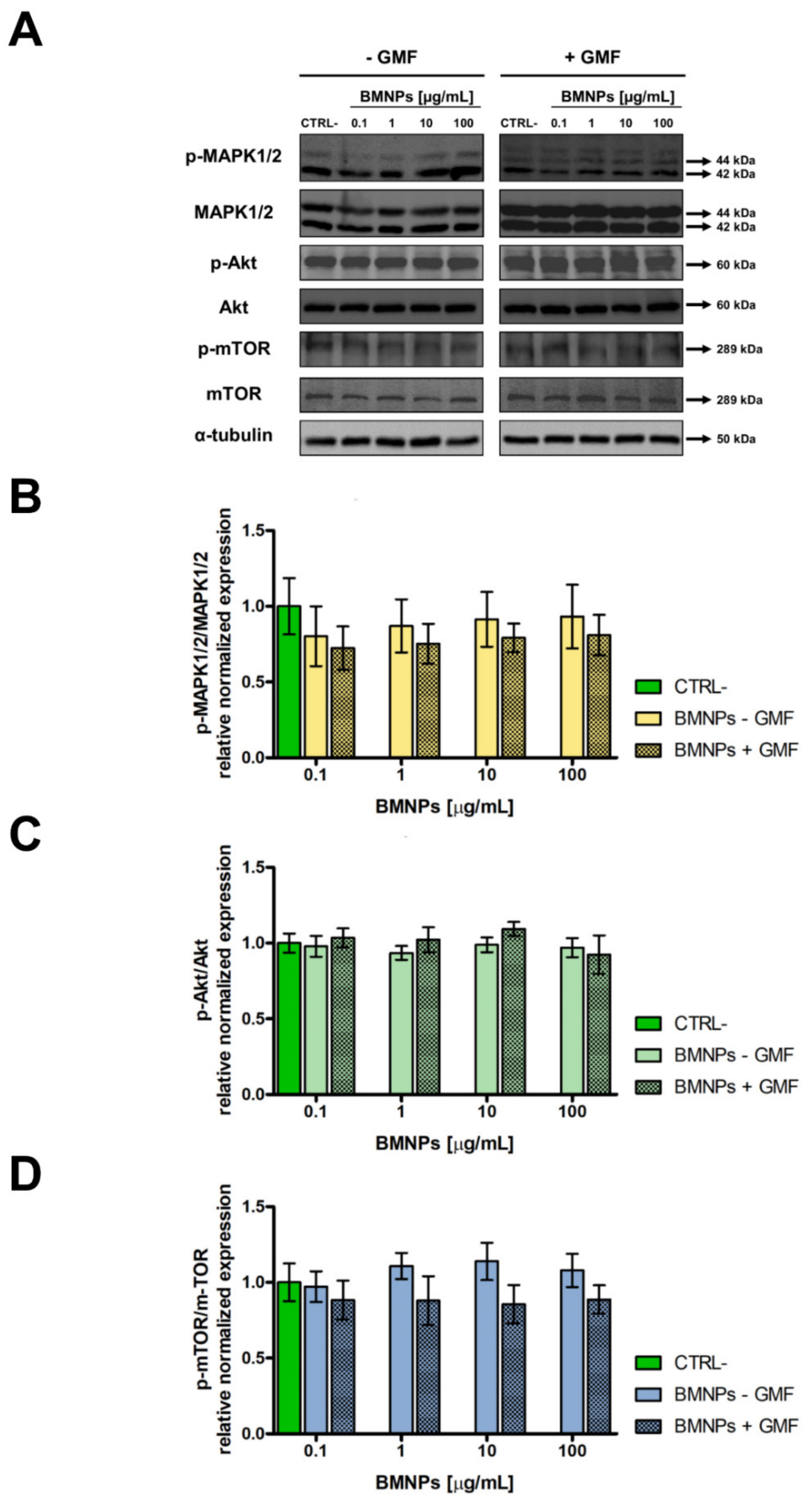

Figure S1. Expression and state of phosphorylation of MAPK1/2 Erk1/2, Akt, and mTOR. (A) Extracts from 4T1 cells incubated for 16h with BMNPs at different concentrations in the absence/presence of a GMF were analyzed in Western blot. All blots shown are representative of three independent experiments. A western blot representative of the bands of tubulin is shown, since in all experiments similar patterns were observed. (B, C, D) Relative densitometric analysis of the bands of the phosphorylated proteins vs the not phosphorylated ones. Data are expressed as the mean ± SD from three independent experiments.

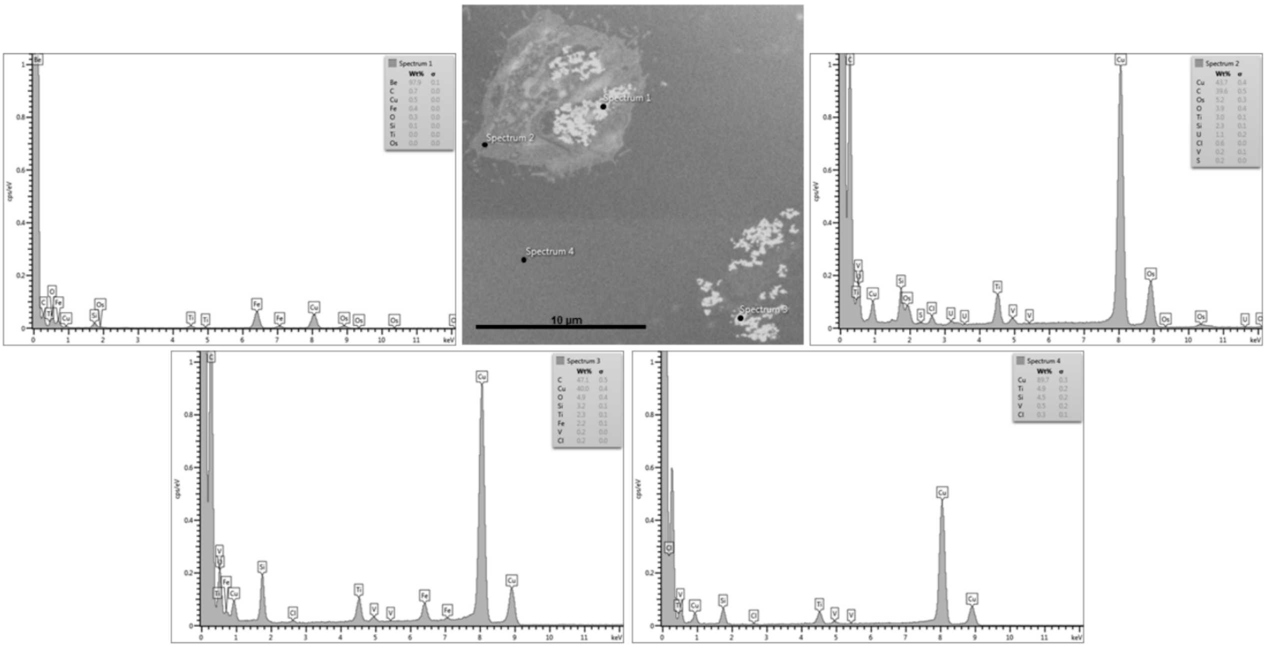

Figure S2. Microanalysis by energy dispersive X-ray (EDX) spectroscopy of 4T1 cells incubated with the 100 µg/mL of BMNPs.

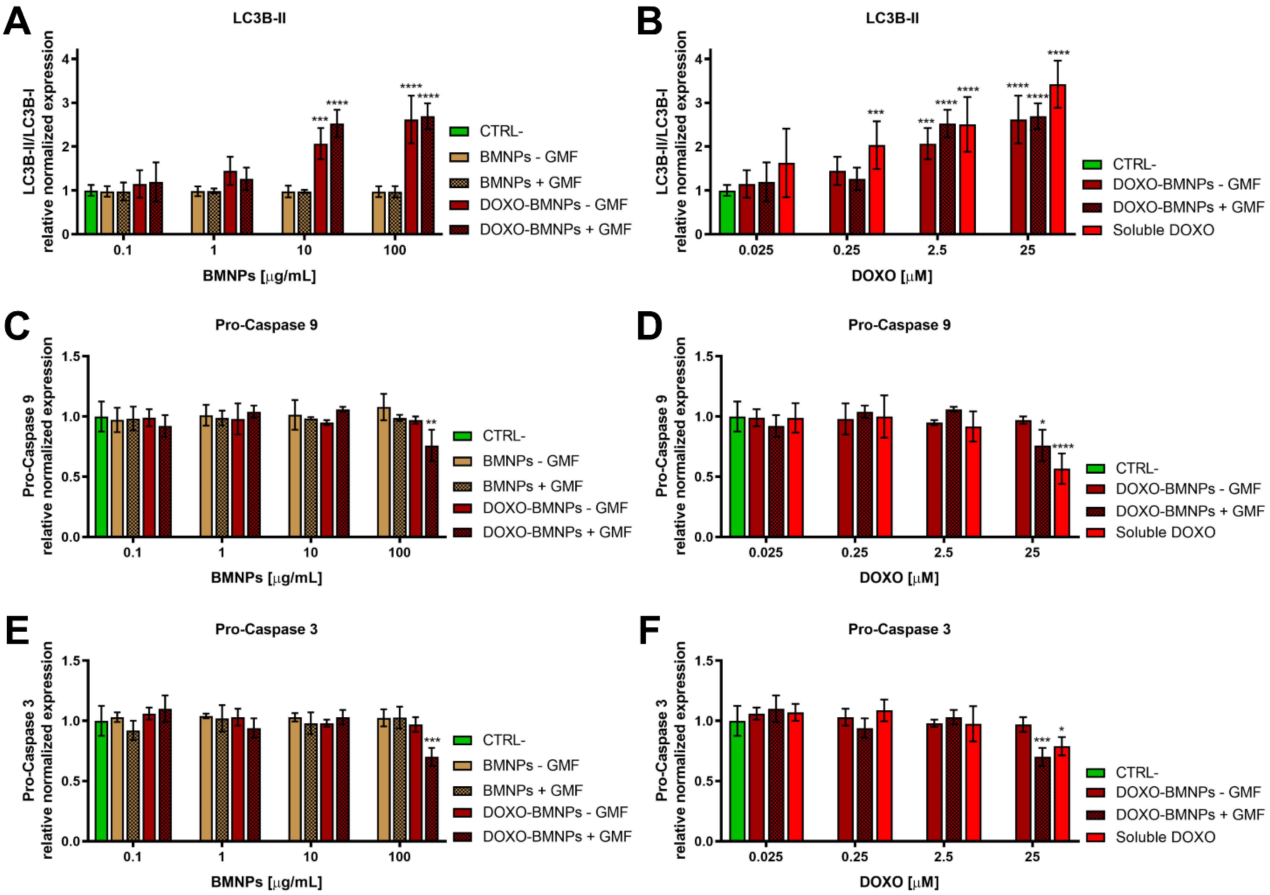

Figure S3. Relative densitometric analysis of the bands of the cleaved/activated LC3B-II vs inactive LC3B-I (A, B), inactive pro-caspase 9 vs active caspase 9 (C, D), and inactive pro-caspase 3 vs active caspase 3 (E, F). Data are referred to the concentration of BMNPs, expressed as µg/mL (A, C, E), , and relative to the amount of DOXO, expressed in µM (B, D, F). Data are expressed as the mean ± SD from three independent experiments. Differences between groups (all compared vs untreated controls)

were assessed by 2way ANOVA with Tukey's multiple comparison post-test (**** p<0.0001; *** p=0.0001; ** p=0.001; * p=0.01).

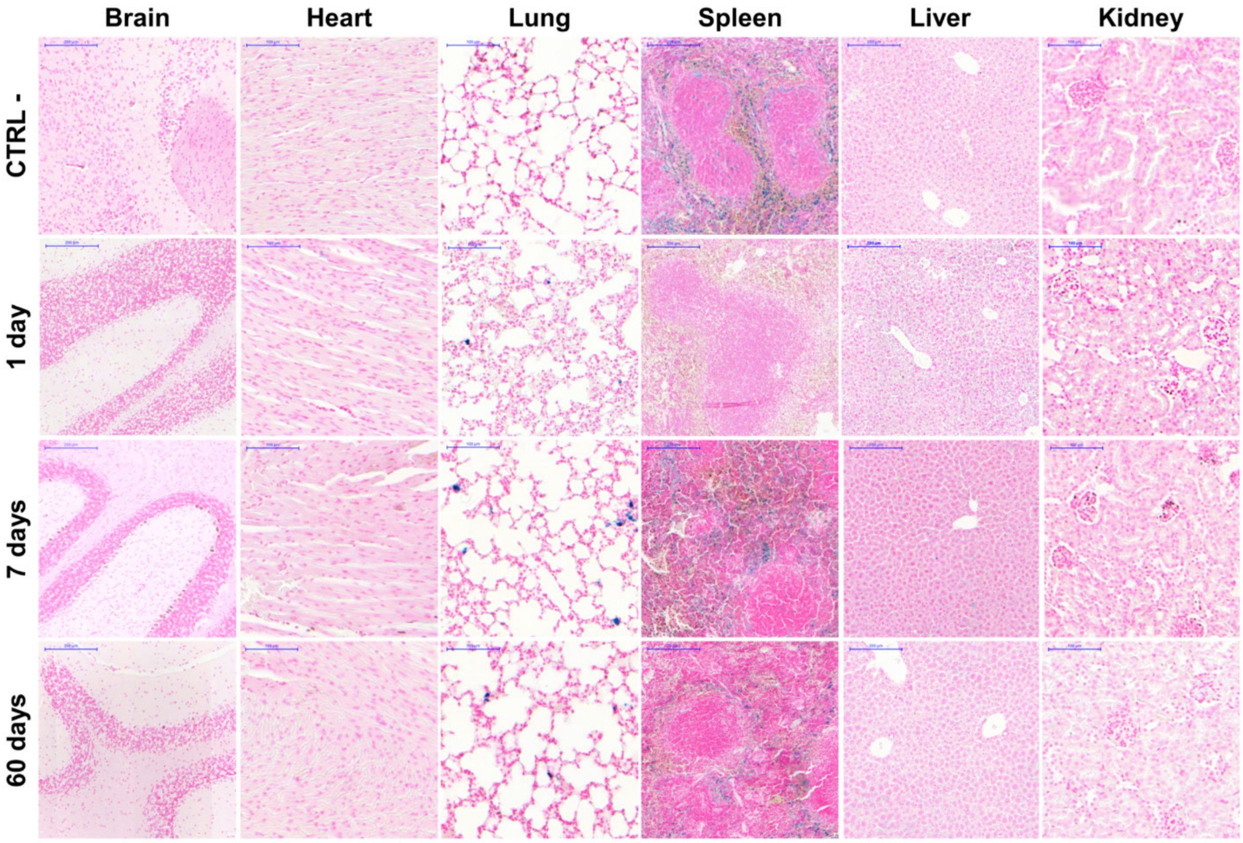

Figure S4. Biodistribution profile of BMNPs after tail-vein injection (10 µg/g mouse body weight) in BALB/c mice (n = 3) grouped into 4 groups differing on the time in which were euthanized (1, 7, and 60 days). Untreated mice were taken as reference samples (bars: =200 µm, brain, spleen, liver; =100 µm, heart, lung, kidney). .

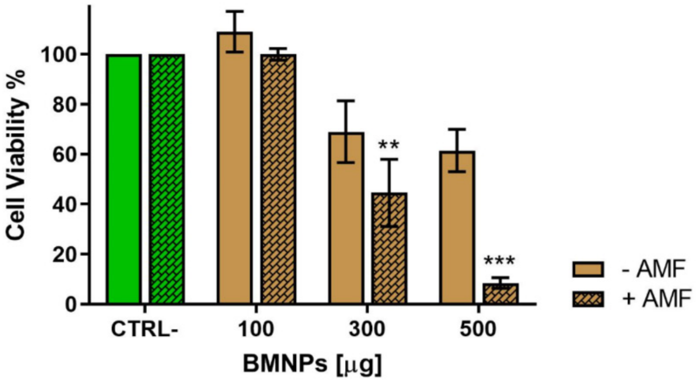

Figure S5. Cytocompatibility/cytotoxicity of BMNPs (100, 300, 500 µg) on 4T1 cells in the absence/presence of an alternating magnetic field (AMF) applied for 20 min. Cell viability was assessed in an MTT carried out for 24h on cells plated after the treatment. Differences between treatments AMF+/- were assessed by 2way ANOVA with Sidak's multiple comparison test (*** p <0.0001; ** p=0.001.

LC3B-I/II

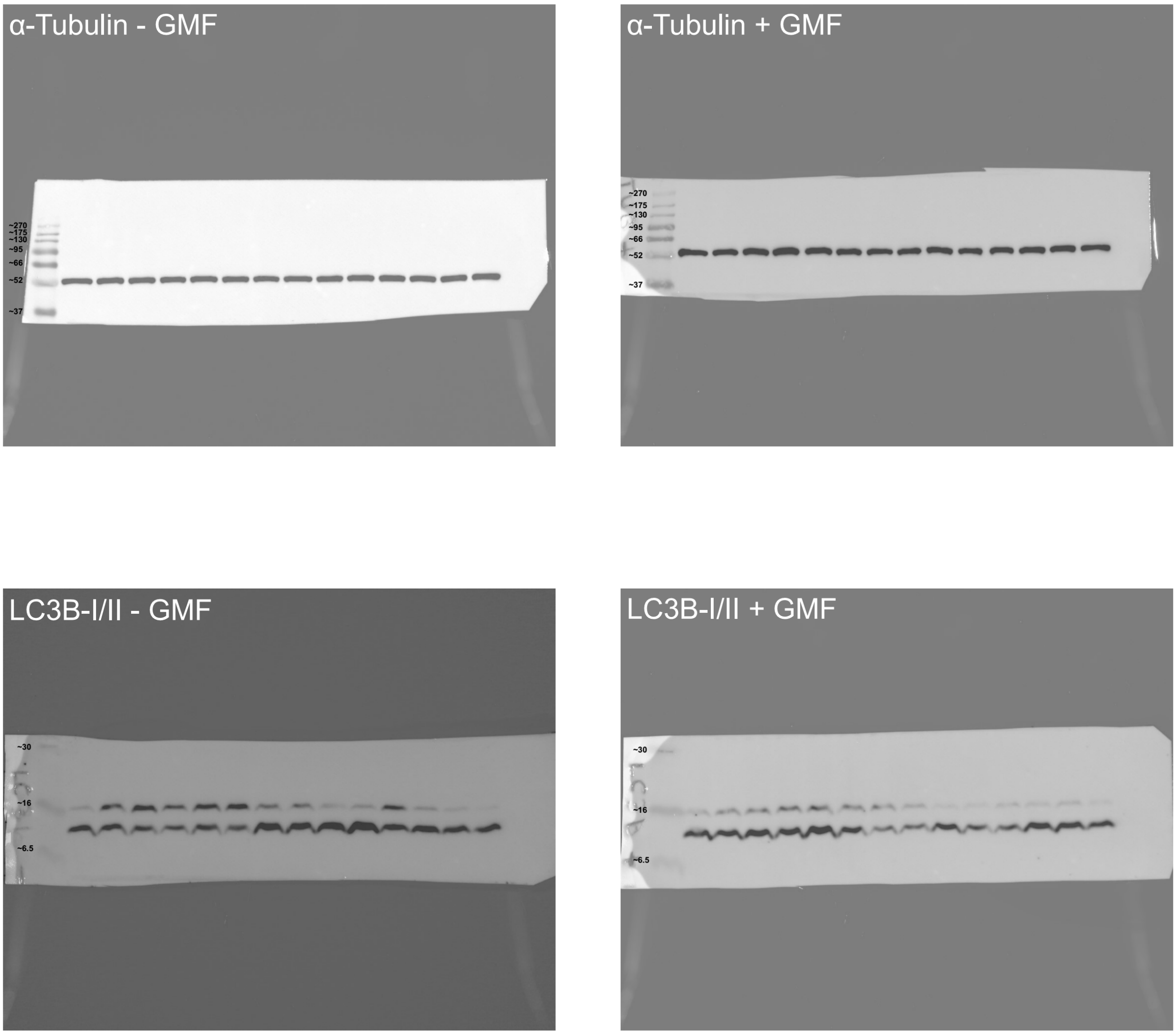

Original Western Blots relative to LC3B-I and LC3B-II -/+ GMF and their relative control α-Tubulin.

These Western Blots of α-Tubulin were used as reference for all the Western Blots shown in Figure 5C, to simplify the figure.

Pro-Caspase 9/Cleaved Caspase 9

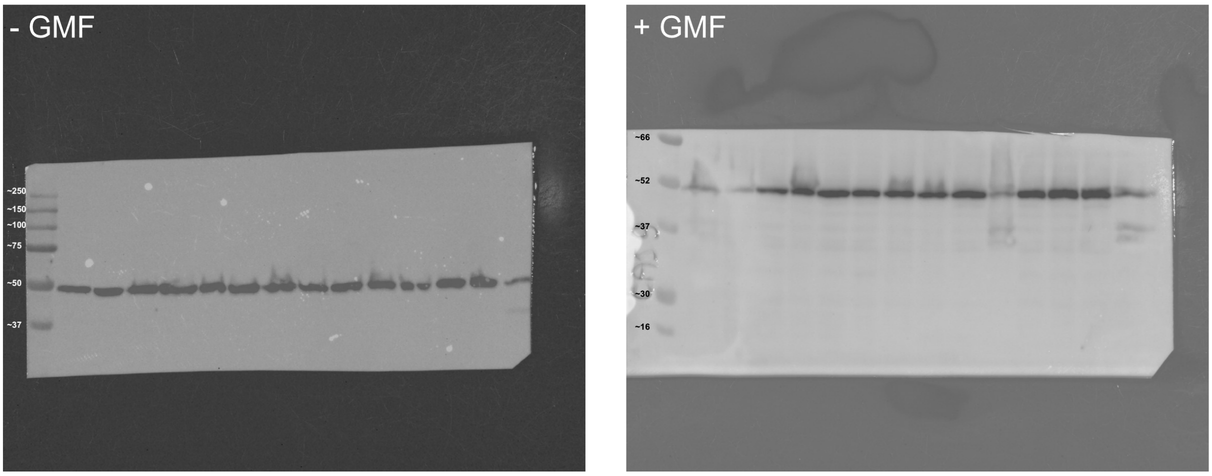

Original Western Blots relative to Pro-Caspase 9 and Cleaved Caspase 9.

The artefact on the left of the right panel is due to the merge with the Molecular Weight (MW) standard. In the Manuscript we put the figure without the merge of the MW.

Note that the MW standard are from two different batches.

Gels run in parallel with the same amounts of cell extract were incubated with the anti-α-Tubulin antibody and showed that similar amounts of proteins were loaded (not shown).

Pro-Caspase 3/Cleaved Caspase 3

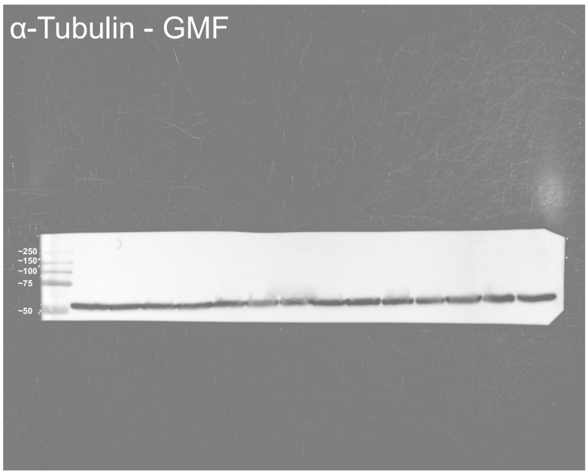

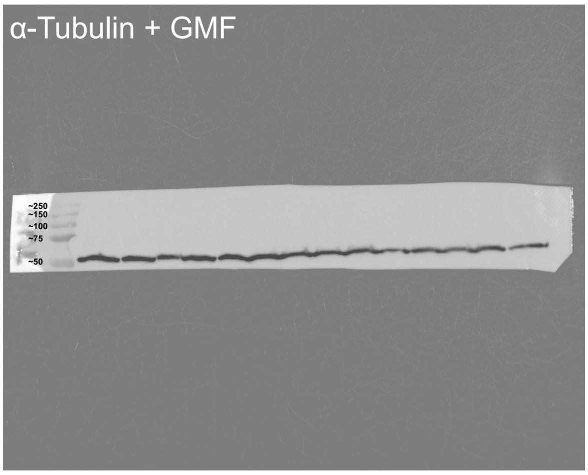

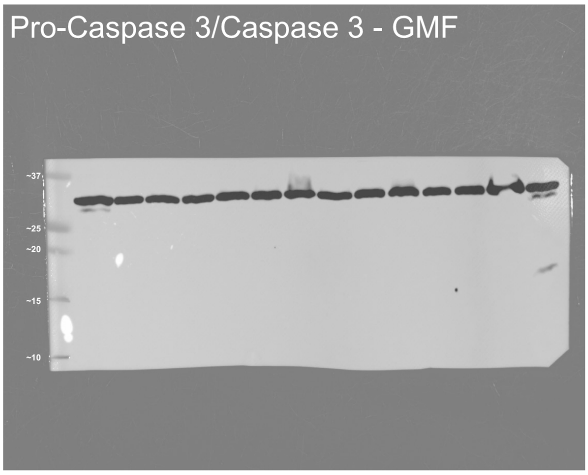

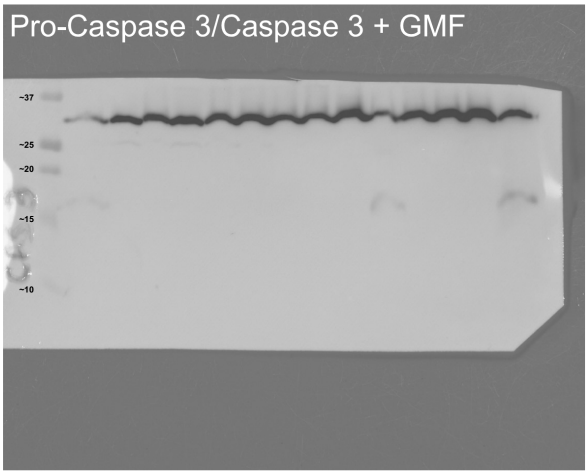

Original Western Blots relative to Pro-Caspase 3 and Cleaved Caspase 3 and their relative control α-Tubulin. Gels run in parallel with the same amounts of cell extract were incubated with the anti-α-Tubulin antibody and showed that similar amounts of proteins were loaded.

p-MAPK1/2 - MAPK1/2

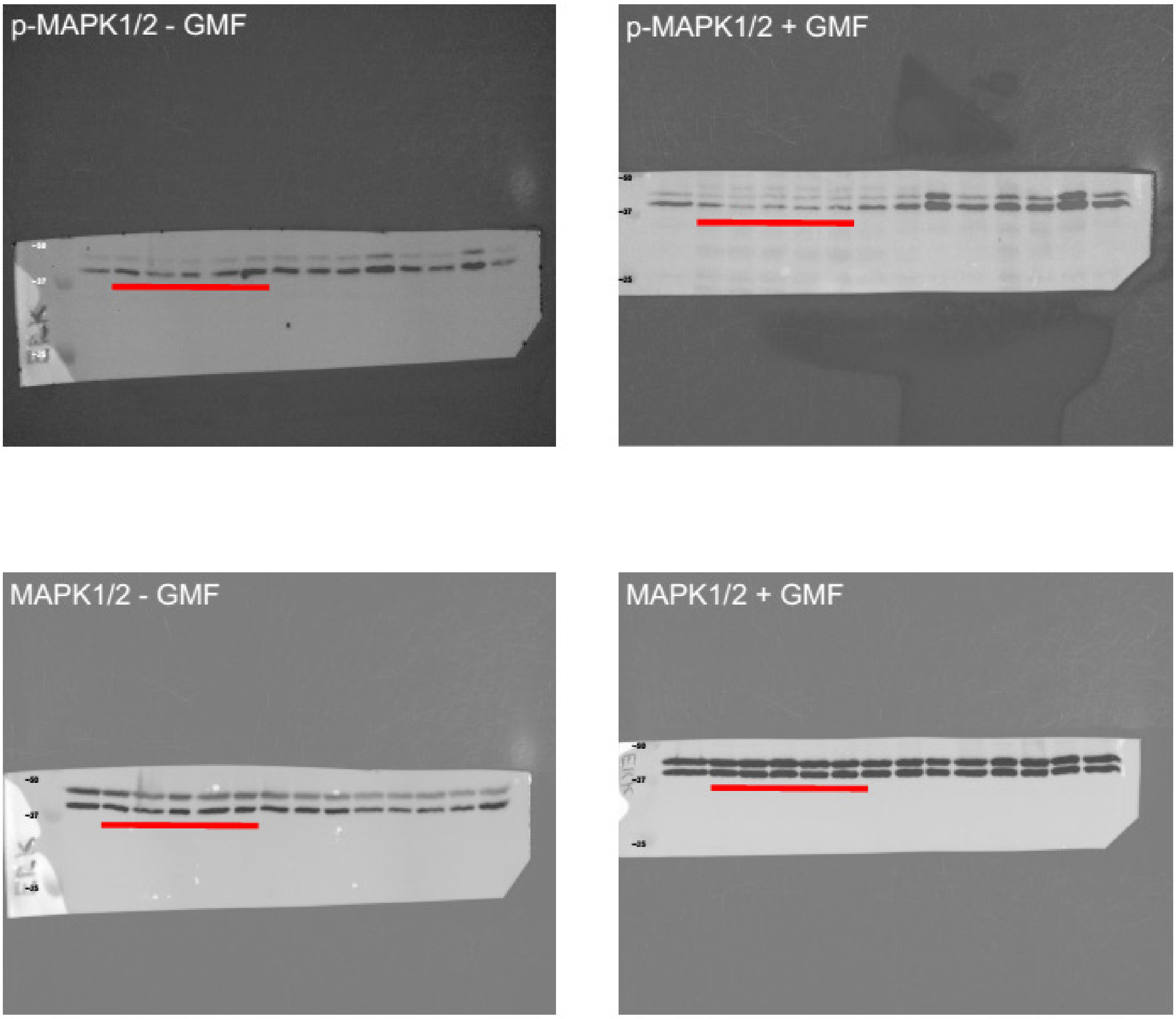

The PVDF paper was first incubated for the visualization of the phosphorylated form; then stripped and incubated again for the total form of the protein. The red lines refer to the five lanes reported in the Supplementary.

p-Akt - Akt

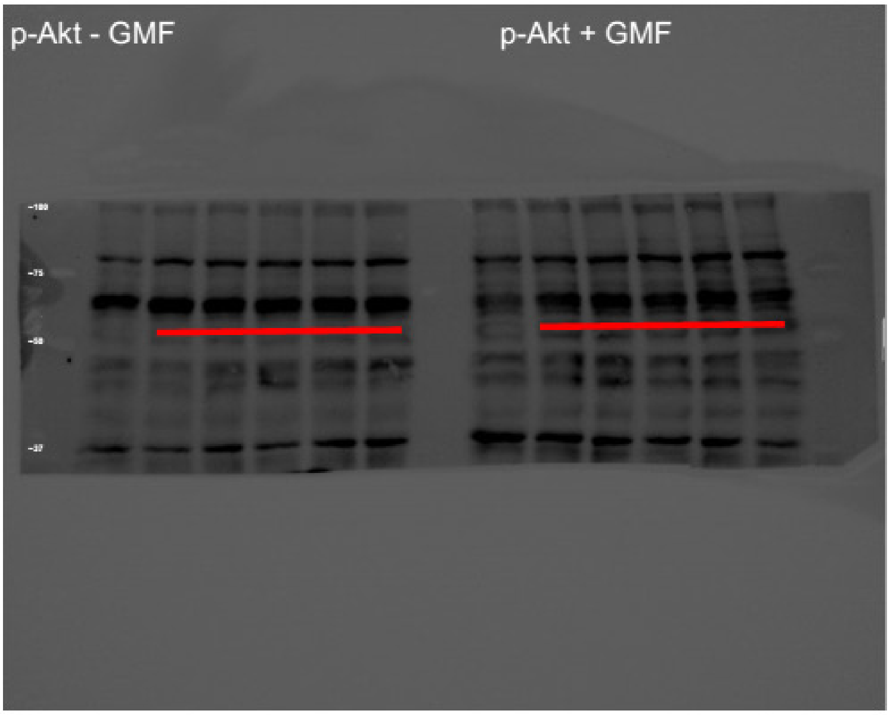

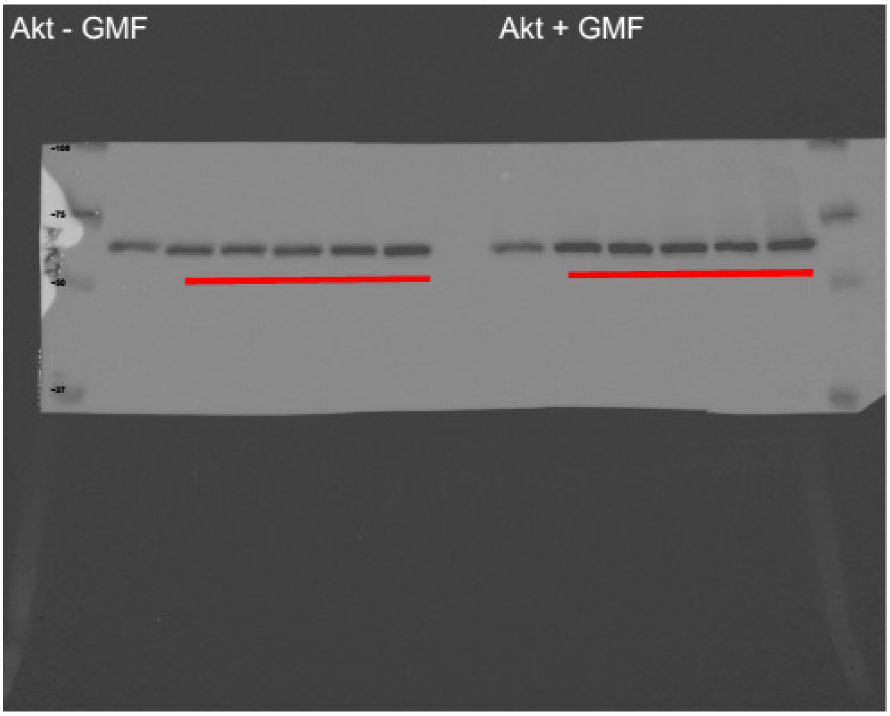

The PVDF paper was first incubated for the visualization of the phosphorylated form; then stripped and incubated again for the total form of the protein. The red lines refer to the five lanes reported in the Supplementary.

p-mTOR - mTOR

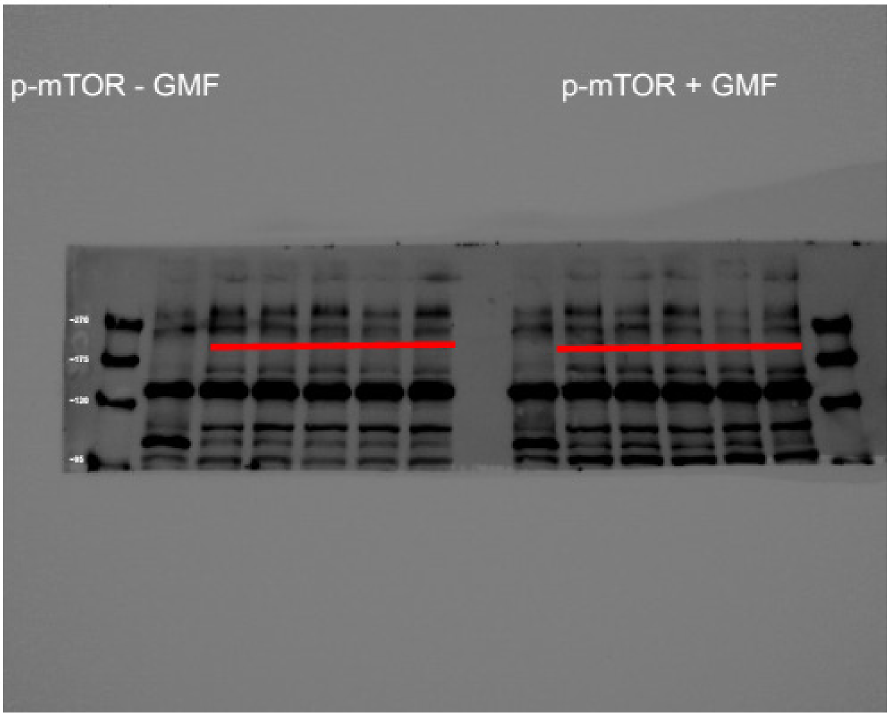

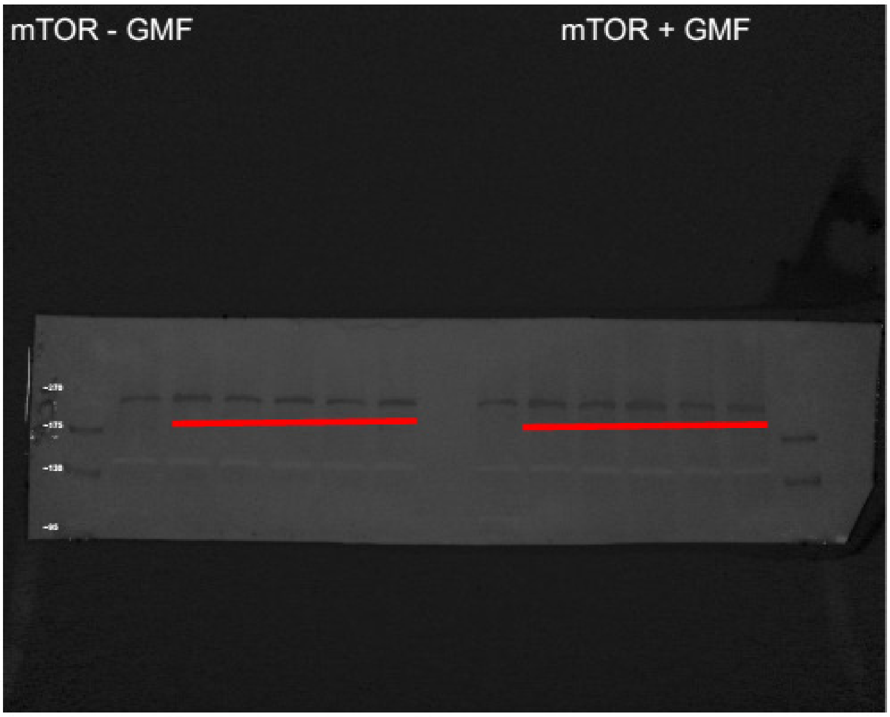

The PVDF paper was first incubated for the visualization of the phosphorylated form; then stripped and incubated again for the total form of the protein. The red lines refer to the five lanes reported in the Supplementary.

α-Tubulin

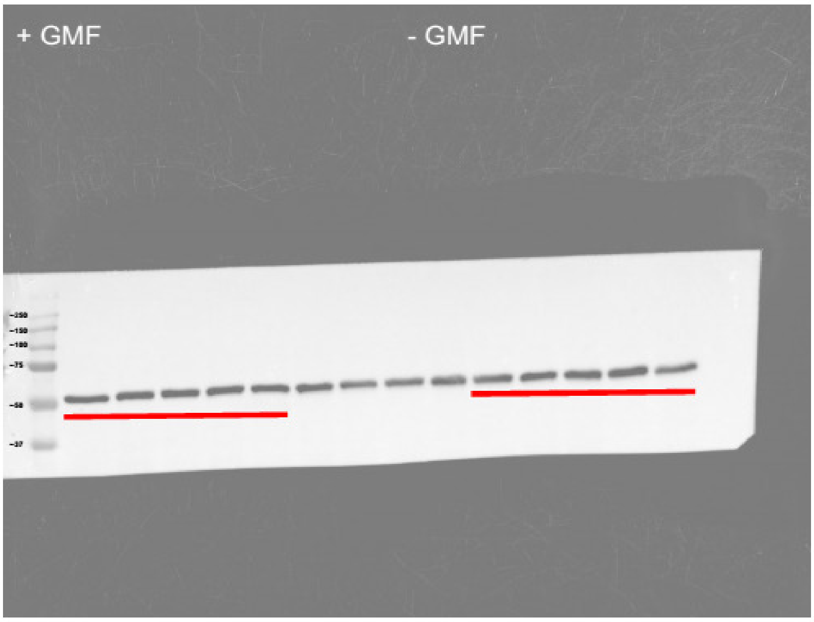

A representative WB for TUBULIN performed from the same extracts run for the analysis of mTOR. Similar results were always obtained for the other protein analysis (MAPK1/2; Akt).

Figure S6. Uncropped Western blots**.**

© 2020 by the authors. Licensee MDPI, Basel, Switzerland. This article is an open access article distributed under the terms and conditions of the Creative Commons Attribution (CC BY) license (http://creativecommons.org/licenses/by/4.0/).
